# Septins organize endoplasmic reticulum-plasma membrane junctions for STIM1-ORAI1 calcium signalling

**DOI:** 10.1038/s41598-019-46862-w

**Published:** 2019-07-25

**Authors:** Zachary B. Katz, Chen Zhang, Ariel Quintana, Björn F. Lillemeier, Patrick G. Hogan

**Affiliations:** 1 Division of Signalling and Gene Expression, La Jolla Institute for Immunology, La Jolla, CA 92037 USA; 2 NOMIS Center for Immunobiology and Microbial Pathogenesis & Waitt Advanced Biophotonics Center, Salk Institute for Biological Studies, La Jolla, CA 92037 USA; 3 Program in Immunology, University of California San Diego, La Jolla, CA 92037 USA; 4 Moores Cancer Center, University of California San Diego, La Jolla, CA 92093 USA; 5 Present Address: Translational Science Division, Clinical Science Department, Moffitt Cancer Center Magnolia Campus, Tampa, FL 33612 USA

**Keywords:** Nanoscale biophysics, Super-resolution microscopy, Signal transduction, Ion transport

## Abstract

ORAI1 Ca^2+^ channels in the plasma membrane (PM) are gated by STIM1 at endoplasmic reticulum (ER)-PM junctions to effect store-dependent Ca^2+^ entry into cells, but little is known about how local STIM-ORAI signalling at junctions is coordinated with overall cellular architecture. Filamentous septins can specify cytoskeletal rearrangements and have been found recently to modulate STIM-ORAI signalling. Here we show by super-resolution imaging of ORAI1, STIM1, and septin 4 in living cells that septins facilitate Ca^2+^ signalling indirectly. Septin 4 does not colocalize preferentially with ORAI1 in resting or stimulated cells, assemble stably at ER-PM junctions, or specify a boundary that directs or confines ORAI1 to junctions. Rather, ORAI1 is recruited to junctions solely through interaction with STIM proteins, while septins regulate the number of ER-PM junctions and enhance STIM1-ORAI1 interactions within junctions. Thus septins communicate with STIM1 and ORAI1 through protein or lipid intermediaries, and are favorably positioned to coordinate Ca^2+^ signalling with rearrangements in cellular architecture.

## Introduction

STIM1 and ORAI1 underlie store-dependent Ca^2+^ influx, a linchpin of local and global cellular signalling and cellular Ca^2+^ balance^1–4^. STIM1 senses a decrease in endoplasmic reticulum (ER)-luminal Ca^2+^ during physiological signalling, undergoes a conformational change, and relocalizes within the ER to specialized sites where the ER is closely apposed to the plasma membrane (PM). There STIM1 recruits and gates plasma membrane ORAI1 channels. ER-PM junctions themselves, through their geometry and protein complement, play a key role in shaping Ca^2+^ signals and integrating Ca^2+^ signalling with other pathways^5–7^. ORAI1 calcium signalling, like other cellular calcium signalling pathways, is intensively modulated by mechanisms that target STIM1 and ORAI1 directly^8,9^. It has been less clear how STIM-ORAI signalling is coordinated with other cellular processes.

Septins 4 and 5, and their homologues in *Drosophila*, have been shown to modulate the efficiency of STIM-ORAI Ca^2+^ signalling^10–12^. Initial evidence suggested that the modulation occurred only at ER-PM junctions— that septins had no effect when the cytoplasmic domain of STIM1, untethered to ER, activated ORAI1 outside of junctions^10^— raising intriguing questions about whether and how septins 4 and 5 could strengthen STIM-ORAI signalling without targeting STIM1 and ORAI1 themselves.

Septins were first studied in budding yeast cell division, where they form a collar around the bud neck that specifies a diffusion barrier for PM proteins and serves as a scaffold to recruit structural and signalling proteins^13,14^. Septins likewise localize at the cleavage furrow in mammalian cells and are required for normal cytokinesis^15–17^, although early embryonic cells and T and B lymphocytes can bypass this requirement^18^. Septin assemblies also demarcate regions in the plasma membrane unrelated to cytokinesis, partitioning the anterior tail compartment of mammalian sperm from the posterior compartment, the membrane of the primary cilium from surrounding plasma membrane, and dendritic spines from dendritic shafts^19–25^. More broadly, septins can exert a profound influence on the cellular architecture and morphogenetic movements of vertebrate and mammalian cells by orchestrating the arrangement of cytoskeletal proteins^26–28^.

ER-PM junctions are small— their typical diameter is less than 200 nm— and so conventional light microscopy lacks the resolution to investigate changes in ORAI1 recruitment to junctions in living cells. Here we have applied single-molecule localization microscopy and single-molecule tracking of ORAI1, septin 4, and STIM1, with the initial goal of investigating whether septins 4/5 specify a corral for ORAI1 at ER-PM junctions. Our results rule out this simple possibility, but delineate several features of PM organization downstream of septins 4/5 that reinforce physiological STIM-ORAI Ca^2+^ signalling. These include the local density of ER-PM junctions, the internal specialization of junctions for STIM-ORAI signalling, and possibly the local modulation of ORAI1 concentration. The links that we demonstrate from septins to cellular Ca^2+^ signalling could help to explain how cells coordinate their local Ca^2+^ responses during physiological signalling with changes in cytoskeletal and PM organization.

## Results

### Single-molecule localization of ORAI1 channels in living cells

We used PATagRFP-ORAI1 and photoactivated localization microscopy (PALM) to examine the distribution and movements of ORAI1 molecules in HeLa cells, at rest and after ER Ca^2+^ store depletion, with an experimentally determined localization uncertainty ~70 nm. The observations of store-depleted cells were made at least 5 min after addition of modified Ringer’s solution containing 1 μM thapsigargin (TG) and 1 mM EGTA, when ORAI1 clusters had formed. STIM1-PAGFP in the TIRF layer served as a marker for near-plasma membrane ER in resting cells and for the more spatially restricted ER-plasma membrane contacts after store depletion.

**Figure 1:**
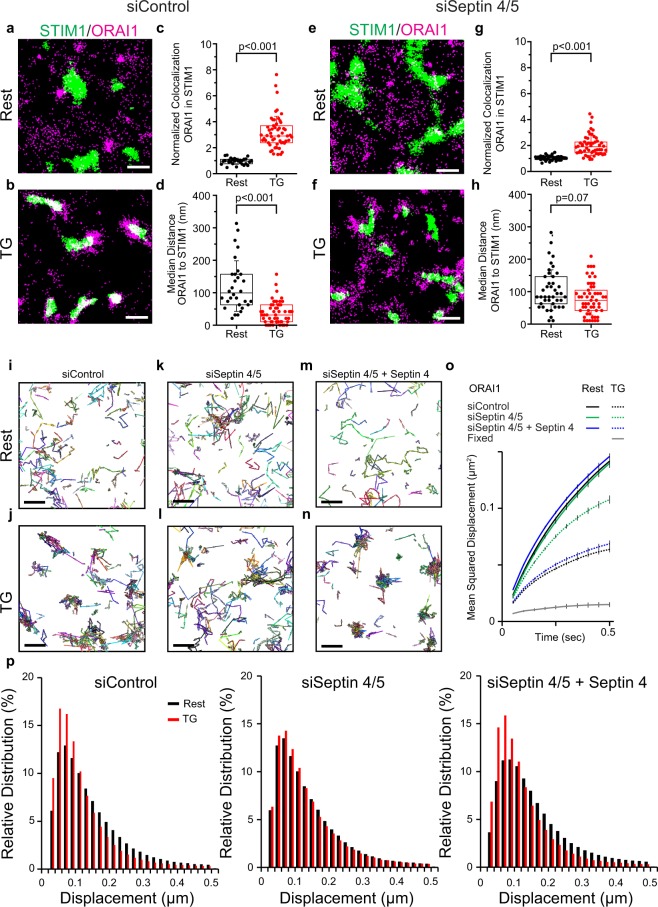
ORAI1 diffusion and recruitment to ER-plasma junctions in siControl- and siSeptin4/5-treated cells. (**a**,**b**) STIM1-PAGFP (green) and PATagRFP-ORAI1 (magenta) were visualized by photoactivated localization microscopy (PALM) in siControl-treated HeLa cells. Representative 3 μm x 3 μm regions are shown from a cell at rest and from a different cell after TG stimulation. (**c**) Normalized colocalization of ORAI1 with STIM1 in individual regions of interest at rest (black) and after TG stimulation (red). The fraction of STIM1 pixels where ORAI1 colocalized has been divided by fraction of all pixels where ORAI1 localized. This normalization avoids inflating the colocalization score merely because more ORAI1 localizations were observed without any preference for colocalization with STIM1. (**d**) Median distance from ORAI1 localizations to the nearest STIM1 territory for individual regions of interest. (**e**–**h**) Images and analysis corresponding to (**a**–**d**) for siSeptin4/5-treated HeLa cells. (**i**–**n**) ORAI1 trajectories in representative 3 μm × 3 μm regions for siControl-treated cells, siSeptin4/5-treated cells, and siSeptin4/5-treated cells expressing RNAi-resistant septin 4, and either at rest or after TG stimulation, as indicated. (**o**) Mean squared displacement (MSD) plots of ORAI1 molecules under the conditions of (**i**–**n**) and in fixed HeLa cells. (**p**) Histograms of apparent ORAI1 displacements in single 50-ms steps for the conditions indicated. Scale bars are 500 nm. (**c**,**d**,**g**,**h**): siControl, data from 7 independent experiments; Rest, 31 regions from 10 cells; TG 56 regions from 18 cells. siSeptin, data from 3 independent experiments; Rest, 42 regions from 14 cells; TG, 42 regions from 14 cells. Statistical significance was determined using a two-tailed *t*-test. (**o**,**p**): siControl, Rest, 57,651 trajectories in 20 cells; siControl, TG, 39,840 trajectories in 20 cells; siSeptin4/5, Rest, 32,993 trajectories in 13 cells; siSeptin4/5, TG, 32,324 trajectories in 13 cells; siSeptin4/5 + Septin4, Rest, 43,713 trajectories in 9 cells; siSeptin4/5 + Septin4, TG, 23,650 trajectories in 10 cells; Fixed, 7764 trajectories in 7 cells. Error bars indicate SEM.

ORAI1 was widely distributed across the footprint of resting cells [Fig. 1a–d; Supplementary Fig. 1]. There was no enrichment of ORAI1 above random in the subset of pixels where STIM1 was localized. (To assess enrichment, we have normalized the observed colocalization to the overall density of ORAI1 localizations in the region analyzed [Fig. 1c, legend]. Normalized colocalization values consistently greater than 1 indicate enrichment, whereas values consistently less than 1 indicate exclusion.) After TG treatment, ORAI1 was recruited to STIM1-marked puncta, with median enrichment above random in the subset of STIM1 pixels ~3, and the median distance from ORAI1 to STIM1 territories was reduced. The normalized colocalization values for stimulated cells were uniformly above those for resting cells. Note that ORAI1 enrichment over random is not the same as the ratio of ORAI1 density at junctions to ORAI1 density outside junctions. We have not attempted to make the latter estimate because, as explained in the next paragraph, ORAI1 scored as ‘nonjunctional’ is likely to include some junctional ORAI1.

The overlap of ORAI1 and STIM1 clusters after TG was imperfect [Fig. 1b]. This result does not imply that ORAI1 clustering occurs without STIM1, because coexpressed STIM1-PATagRFP and STIM1-PAGFP examined in the same way showed similarly imprecise overlap [Supplementary Fig. 2]. Both biological and technical considerations probably contributed to the imperfect overlap. On the biological side, ER-PM junctions are not static at 37 C, and small local redeployments of individual junctions between the acquisitions of STIM1 and ORAI1 images, which were sequential, would affect apparent overlap. On the technical side, ThunderSTORM localization software may fail to map all STIM1 or ORAI1 molecules in areas of very high density, a shortcoming it shares with other localization algorithms; and, conversely, limited sampling may lead to false negative pixels at loci where STIM1 or ORAI1 are sparsely distributed.

We examined the effect of TG on ORAI1 localization in siSeptin4/5-treated cells. ORAI1 was still measurably recruited to junctions marked by STIM1 in these cells, but recruitment was less pronounced than in control cells, with median enrichment above random ~2, and an apparent small decrease in the median ORAI1-STIM1 distance was not statistically significant [Fig. 1e–h]. The findings replicated lower-resolution observations by conventional TIRF microscopy^10^ and supported the use of single-molecule localization techniques for detailed analysis of septin 4/5 effects on STIM-ORAI signalling.

### Effects of septin 4/5 depletion on ORAI1 trajectories

ORAI1 localizations in sequential 50-ms frames can be linked to display the trajectories of individual ORAI1 channels [Fig. 1i–n; Supplementary Movies 1–4] for quantitative analysis of ORAI1 movements. One frequently used method of analysis is the mean squared displacement (MSD) plot [Fig. 1o]. MSD plots of ORAI1 channel movements mirrored the observations from ORAI1 localizations in Fig. 1a–h. ORAI1 trajectories in control cells were, on average, more confined after TG, as indicated by the early flattening of the MSD curve. TG-induced confinement was less pronounced in siSeptin4/5-treated cells, but reconstitution with RNAi-resistant septin 4 restored it, firmly linking the confinement to a direct or indirect effect of septins.

MSD analysis can be particularly informative regarding diffusive movements. Since the ORAI1 MSD plots exhibited curvature within the first 200 ms, indicative of spatial restrictions on ORAI1 diffusion on that time scale, we could not take the usual approach of estimating an average diffusion coefficient *D* for each condition as a linear fit to *r*^2^ values over several steps. Instead, we applied two alternative methods to estimate average *D*: from single-step *r*^2^ values, and from the slope of the MSD plot between 50 ms and 100 ms [Table 1]. Corrections for localization uncertainty and for blurring due to particle movement within each frame have to be introduced in the single-step calculation^29–32^ [see Methods]. No corrections are required in the 50ms–100 ms slope calculation, since localization error and blurring make the same average contributions to the 50 ms and 100 ms *r*^2^ values, and therefore the corrections cancel upon subtraction. The two independent estimates of the ORAI1 diffusion coefficient agreed for all conditions observed [Table 1].

**Table 1:** Diffusion estimates for ORAI1 from single molecule tracking datasets.

ORAI1	D [est1] µm^2^/sec	D [est2] µm^2^/sec
siControl Rest	0.112 ± 0.001	0.104 ± 0.001
siControl TG	0.058 ± 0.001	0.056 ± 0.001
siSeptin 4/5 Rest	0.103 ± 0.002	0.101 ± 0.003
siSeptin 4/5 TG	0.093 ± 0.002	0.086 ± 0.003
siSeptin 4/5 + Septin 4 Rest	0.146 ± 0.002	0.111 ± 0.002
siSeptin 4/5 + Septin 4 TG	0.067 ± 0.002	0.058 ± 0.002

The quantitative analysis bore out the qualitative conclusions drawn above. The average *D* value in resting HeLa cells, ~0.11 μm^2^ s^−1^ [Table 1], was similar to the value measured in HEK293 cells^33^. Store depletion elicited an approximate 50% reduction in average *D*. ORAI1 mobility in resting siSeptin 4/5-treated cells resembled ORAI1 mobility in resting control cells, with average diffusion coefficient ~0.10 μm^2^ s^−1^, but diffusion was on average much less affected by calcium store depletion. TG-triggered slowing of ORAI1 was restored by reconstitution of siSeptin4/5-treated cells with RNAi-resistant septin 4.

The effects of siSeptin4/5 and of septin 4 reconstitution were equally evident in histograms depicting the 50-ms, 100-ms, or 150-ms step distances observed in the raw data [Fig. 1p for the 50-ms interval; Supplementary Fig. 3 for all three time intervals]. This alternative view offers richer detail than the averaged diffusion coefficients or the averaged displacements of an MSD plot. Collectively, the data of Fig. 1 establish that the slowed diffusion of ORAI1 channels and their confinement to ER-PM junctions after store depletion are compromised by a reduction of septins 4/5 in the cell.

### Septin 4/5 depletion has no effect on STIM1 trajectories in STIM-ORAI coexpressing cells

**Figure 2:**
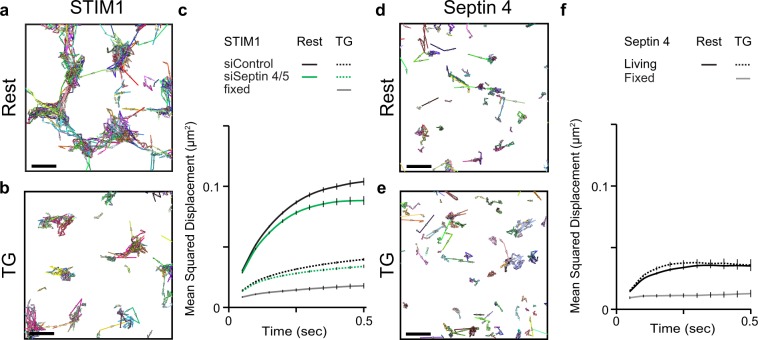
STIM1 and septin 4 diffusion in HeLa cells. (**a**,**b**) STIM1-PATagRFP trajectories for siControl-treated cells, at rest or after TG stimulation, as indicated. (**c**) MSD plots of STIM1 molecules under the conditions indicated. (**d**,**e**) PATagRFP-septin 4 trajectories at rest or after TG stimulation. (**f**) MSD plots of septin 4 molecules under the conditions indicated. Scale bars are 500 nm. (**c**): siControl, Rest, 42,528 trajectories in 18 cells; siControl, TG, 62,057 trajectories in 28 cells; siSeptin4/5, Rest, 33,621 trajectories in 19 cells; siSeptin4/5, TG, 45,865 trajectories in 27 cells; Fixed, 11,436 trajectories in 13 cells. Error bars indicate SEM. (**f**): Living, Rest, 22,882 trajectories in 24 cells; Living, TG, 13,578 trajectories in 19 cells; Fixed, 4683 trajectories in 6 cells. Error bars indicate SEM.

We followed STIM1-PATagRFP trajectories similarly, in cells coexpressing ORAI1 to maintain consistency in the experimental conditions [Fig. 2]. The trajectories in resting cells had a pattern consistent with STIM1 diffusion within ER tubules located in the TIRF layer. An average *D* value calculated from single-step *r*^2^ values, 0.16 μm^2^ s^−1^ [Table 2], was in line with an estimate from single-molecule measurement in HEK293 cells and with earlier estimates from FRAP measurements^33–35^. The flattening of the MSD plot at longer times probably reflects movement of the more mobile STIM1 molecules out of the TIRF layer^33^. STIM1 diffusion was slowed and STIM1 trajectories became more confined after store depletion. Although the STIM1 MSD plot for TG-treated cells did not reach a clear plateau within 500 ms, its shape suggested an equivalent confinement radius less than 250 nm [Supplementary Fig. 4], in line with the expected dimensions of ER-PM junctions in HeLa cells. In contrast to the marked effect of septin 4/5 depletion on ORAI1 movements, septin 4/5 depletion did not alter STIM1 diffusion appreciably either in resting cells or after TG treatment. This observation assumed importance in the later analysis of ORAI1 retention at junctions.

**Table 2:** Diffusion estimates for STIM1 and Septin 4 from single molecule tracking datasets.

STIM1	D [est1] µm^2^/sec	D [est2] µm^2^/sec
siControl Rest	0.162 ± 0.002	0.107 ± 0.002
siControl TG	0.041 ± 0.001	0.037 ± 0.001
siSeptin 4/5 Rest	0.150 ± 0.002	0.101 ± 0.003
siSeptin 4/5 TG	0.037 ± 0.001	0.033 ± 0.001
**Septin 4**	**D [est1] µm** ^**2**^ **/sec**	**D [est2] µm** ^**2**^ **/sec**
Rest	0.044 ± 0.002	0.051 ± 0.002
TG	0.049 ± 0.003	0.060 ± 0.003

### Septin 4 territories are largely distinct from ER-PM junctions

PATagRFP-septin 4 at the plasma membrane exhibited highly restricted mobility [Fig. 2]. The MSD plot plateau of ~0.035 μm^2^ translates, after correction for localization uncertainty, to an equivalent confinement radius (*r*_c_) of ~160 nm both in resting cells and after store depletion. The value is similar to that determined for the septin Cdc12-GFP in septin bundles associated with the plasma membrane of the fungus *Ashbya gossypii*^36,37^, suggesting that septin 4 is incorporated into a larger septin framework at the HeLa cell PM. Consistent with this interpretation, PAGFP-septin 4 localizations, in aggregate, defined territories that were relatively stable on the time scale of our experiments [Supplementary Fig. 5; and see Methods].

**Figure 3:**
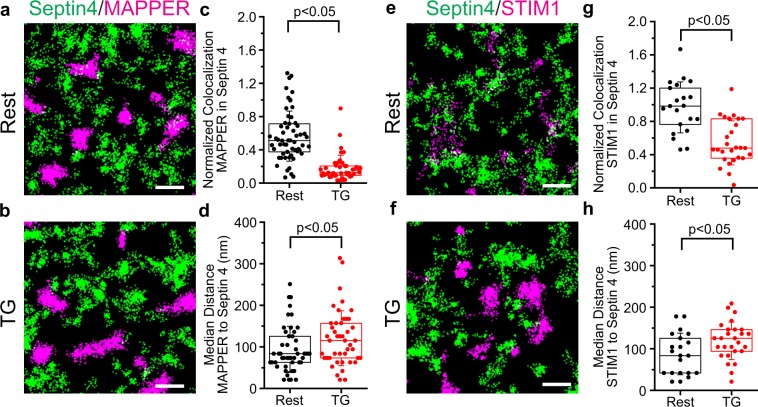
Septin 4 territories segregate from ER-PM junctions both in resting cells and after ER calcium depletion. (**a**,**b**) Localizations of PAGFP-septin 4 (green) and PATagRFP-MAPPER (magenta), which demarcates ER-PM junctions, in a cell at rest and in a different cell after TG stimulation. (**c**) Normalized colocalization of MAPPER with septin 4 in individual regions of interest at rest (black) and after TG stimulation (red). (**d**) Median distance from MAPPER localizations to the nearest septin territory for individual regions of interest. (**e**–**h**) Corresponding data for PAGFP-septin 4 and STIM1-PATagRFP. Examples of septin 4 movement relative to STIM1 are shown in Supplementary Movies 9 and 10. Scale bars are 500 nm. (**c,d**,**g**,**h**): Septin4/MAPPER, data from 2 independent experiments with more than 40 cells; Rest, 58 regions; TG 45 regions. Septin4/STIM1, data from 2 independent experiments; Rest, 21 regions from 7 cells; TG, 27 regions from 9 cells. Statistical significance was determined using a two-tailed *t*-test.

We mapped the plasma membrane distribution of PAGFP-septin 4 relative to ER-PM junctions, using PATagRFP-MAPPER, an engineered marker of close ER-PM contacts^38^, and STIM1-PATagRFP as alternative markers for ER-PM junctions. Septin 4 territories were largely distinct from junctions both in resting cells and after TG treatment [Fig. 3]. This separation was visible qualitatively in both conditions for MAPPER, and quantitatively the median normalized colocalization of MAPPER with septin 4 was appreciably less than 1 [Fig. 3a–d; Supplementary Movies 5, 6]. Likewise, STIM1 segregated from septin 4 when it relocalized to junctions after store depletion with TG [Fig. 3e–h; Supplementary Movies 7, 8]. The median normalized colocalization of STIM1 with septin 4 decreased from ~1 in resting cells, indicating a random distribution of STIM1 relative to septin 4, to ~0.5 following store depletion. The median distance from STIM1 or MAPPER to the nearest septin 4 territory increased after store depletion [Fig. 3d,h], probably reflecting the previously observed reorganization of septins around junctions^10^ in addition to the relocalization of STIM1. The separation of septin 4 from junctions was clear in views across entire cell footprints [Supplementary Fig. 6]. Thus, strikingly, a substantial patchwork of structures incorporating septin 4 and a large number of ER-PM junctions are interleaved at the plasma membrane, but the two occupy disjoint areas of the membrane.

### Septins 4/5 do not direct ORAI1 to ER-PM junctions

**Figure 4:**
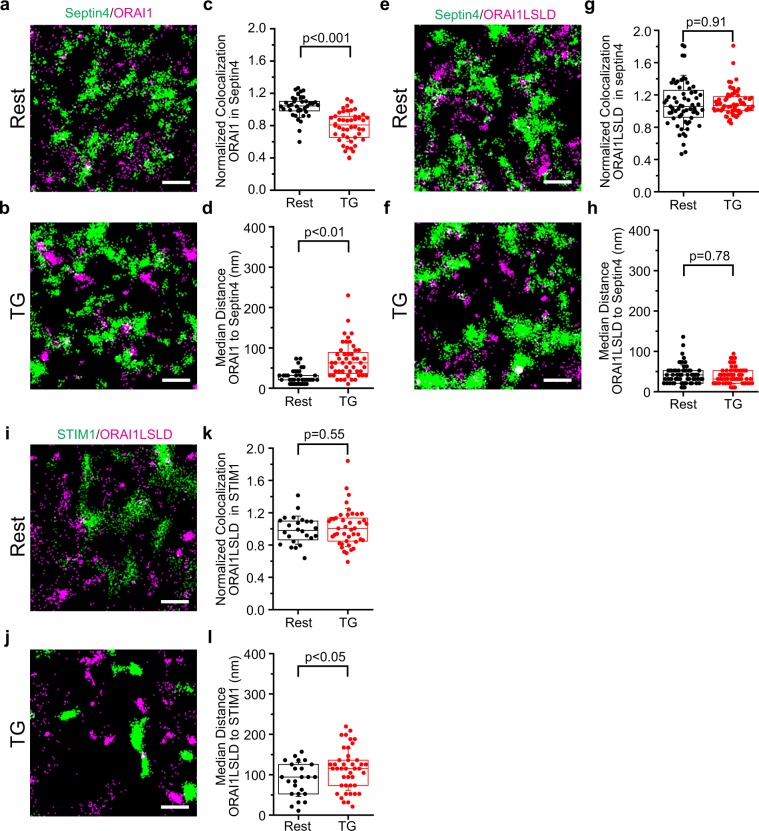
Localization of ORAI1 and ORAI1-LSLD relative to septin territories. (**a**,**b**) Localizations of PAGFP-septin 4 (green) and PATagRFP-ORAI1 (magenta), in a cell at rest and in a different cell after TG stimulation. (**c**) Normalized colocalization of ORAI1 with septin 4 in individual regions of interest at rest (black) and after TG stimulation (red). (**d**) Median distance from ORAI1 localizations to the nearest septin territory for individual regions of interest. (**e–h**) Corresponding data for PAGFP-septin 4 and PATagRFP-ORAI1(L273S/L276D). Examples of ORAI1 and ORAI1-LSLD movement relative to septin 4 are shown in Supplementary Movies 13–16. (**i**–**l**) The experiment corresponding to Fig. 1(a–d) for ORAI1(L273S/L276D), showing that the mutant ORAI1 protein is not recruited to ER-PM junctions after TG treatment. Scale bars are 500 nm. (**c**,**d**,**g**,**h**,**k**,**l**): Septin4/ORAI1, data from 3 independent experiments; Rest, 38 regions from 12 cells; TG, 48 regions from 12 cells. Septin4/ORAI1LSLD, data from 3 independent experiments; Rest, 65 regions from 21 cells; TG, 68 regions from 22 cells. STIM1/ORAI1LSLD, data from 2 independent experiments; Rest, 24 regions from 8 cells; TG, 42 regions from 14 cells. Statistical significance was determined using a two-tailed *t*-test.

The sharp separation between septin 4 territories and ER-PM junctions raised the possibility that exclusion of ORAI1 from septin territories could assist in directing ORAI1 to junctions. As a first approach to this question, we examined the localization of ORAI1 relative to PAGFP-septin 4 [Fig. 4a–d]. ORAI was indifferent to septin 4 territories in resting cells (median normalized colocalization ~1), ruling out constitutive ORAI1 exclusion from the territories, but redistributed away from septin 4 after calcium store depletion (median normalized colocalization ~0.8). Of course, given the results of the previous section, ORAI1 might be expected to move away from septin 4 simply because a fraction of ORAI1 becomes confined at junctions after store depletion. Therefore, we constructed an ORAI1 channel, ORAI1-LSLD, that does not interact with STIM1 by incorporating the previously reported mutations L273S and L276D, either of which alone abrogates the STIM1-ORAI1 interaction^39,40^. In measurements similar to those made for wildtype ORAI1, ORAI1-LSLD did not relocalize away from septin 4 following store depletion [Fig. 4e–h]. We conclude that wildtype ORAI1 segregates from septin 4 only due to its STIM1-dependent partial confinement at junctions after store depletion.

Further consideration of our ORAI1-LSLD localization data and data from the single ORAI1(L273S) and ORAI1(L276D) mutants^39,40^ supports an additional strong conclusion. Provided the LSLD mutations affect only the interaction with STIM proteins, the fact that ORAI1-LSLD is not preferentially localized at junctions after store depletion [Fig. 4i–l; Supplementary Movies 11, 12] rules out any recruitment of ORAI1 channels to junctions by mechanisms other than their interaction with STIM proteins. The argument applies to any hypothesized STIM-independent mechanism, whether it involves septins or not.

### Septins 4/5 enhance ORAI1 retention at junctions

**Figure 5:**
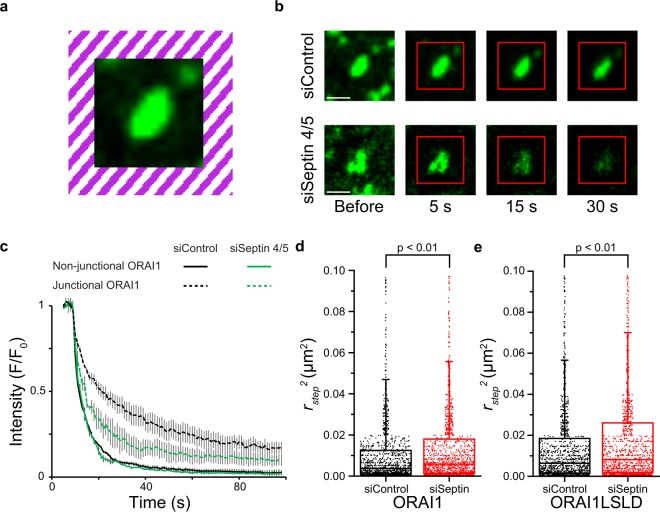
ORAI1 diffusion away from junctions in siControl- and siSeptin4/5-treated cells. (**a**) Design of the modified inverse FRAP experiment. A bleaching laser illuminating the hatched area was turned on at time 0 and remained on subsequently, except during image collection at 1 s intervals. (**b**) Representative images of ORAI1 clusters in siControl-treated and siSeptin4/5-treated cells at times indicated relative to the onset of bleaching laser illumination. The inner boundary of the bleached region is marked. Scale bars are 1 μm. (**c**) Decay curves of ORAI1 fluorescence in regions including ORAI1 clusters after TG stimulation (dashed lines) and in regions of comparable size in resting cells (solid lines), for siControl cells (black lines) and siSeptin 4/5 cells (green lines). (**d**) First-step *r*^2^ values for wildtype ORAI1 trajectories starting within STIM1-marked junctions, in siControl-treated and siSeptin4/5-treated cells, after TG stimulation. The population-average *r*^2^ values used to estimate the average diffusion coefficient *D* of wildtype ORAI1 were, respectively, 0.0147 μm^2^ and 0.0191 μm^2^. (**e**) First-step *r*^2^ values for ORAI1-LSLD trajectories starting within STIM1-marked junctions. The population-average *r*^2^ values used to estimate the average diffusion coefficient *D* of ORAI1-LSLD were 0.0197 μm^2^ and 0.0258 μm^2^. Outlying data points with *r*_step_^2^ exceeding ~0.1 μm^2^ have been omitted in (**d**) and (**e**). (**c**): siControl, TG, n = 3; siSeptin4/5, TG, n = 3; siControl, Rest, n = 3; siSeptin4/5, Rest, n = 3. Error bars indicate SEM. (**d**,**e**): *n* = 1500 trajectories for each condition. Statistical significance was determined using a two-tailed *t*-test.

In a different mechanism compatible with the evidence that STIM-ORAI interaction is required for ORAI1 recruitment to junctions, septins 4/5 might enhance the retention of ORAI1 channels at junctions after their STIM-dependent clustering. We used modified inverse FRAP to look directly at the retention of GFP-ORAI1 at individual ER-plasma membrane junctions of stimulated cells. Following cluster assembly, the region surrounding an individual ORAI1 cluster was continuously illuminated at high laser intensity, except during image acquisition, so that any ORAI1 leaving the cluster would be bleached and any ORAI1 newly added to the cluster would be nonfluorescent [Fig. 5a]. Under these conditions, most of the junctional ORAI1 signal disappeared over a period of tens of seconds in both control and septin 4/5-depleted cells [Fig. 5b,c]. However, the fluorescence loss was more rapid in septin-depleted cells, and left a lower fraction of relatively stable signal at times from 50–80 s after the onset of bleaching laser illumination. The residual signal at 10 s and 80 s in control cells was 50% and 16%, respectively, whereas the residual signal at 10 s and 80 s in siSeptin4/5 cells was 30% and 9%. In control experiments, ORAI1 loss from regions of comparable size in resting cells was very rapid and was unaltered by septin depletion [Fig. 5c].

Part of the explanation of the inverse FRAP results is that a smaller fraction of junctional ORAI1 is engaged with STIM1 when septins 4/5 have been depleted. This is evident in higher ORAI1 mobility in septin 4/5-depleted cells than in control cells, in trajectories starting within STIM1-marked junctions [Fig. 5d]. The average ORAI1 diffusion coefficient at junctions after TG, calculated as in Table 1 from single-step *r*^2^ values [see Methods], was 0.080 μm^2^ s^−1^ in siSeptin4/5-treated cells, 0.047 μm^2^ s^−1^ in siControl cells. Since STIM1 visible in the TIRF layer after TG is essentially confined to junctions, STIM1 mobility under these conditions is adequately measured from overall MSD plots, where single-step *D* estimates were in the range 0.038–0.041 μm^2^ s^−1^ [Table 2]. Thus siSeptin4/5-treated cells show an especially marked disparity between ORAI1 and STIM1 diffusion. With the simplifying assumption that ORAI1 has only two diffusional states, as free ORAI1 or as STIM1-bound ORAI1, and taking the diffusion parameters for ORAI1-LSLD from single-step measurements at junctions [Fig. 5e] as representative of the diffusion of free wildtype ORAI1, we estimated the respective fractions of ORAI1 bound as 84% and 56% [see Methods]. This analysis shows that septins 4/5 enhance the retention of ORAI1 at individual ER-PM junctions of store-depleted cells, and do so mainly by promoting the effective engagement of ORAI1 with STIM1.

### TG/EGTA treatment increases PM barriers to ORAI1 diffusion

**Figure 6:**
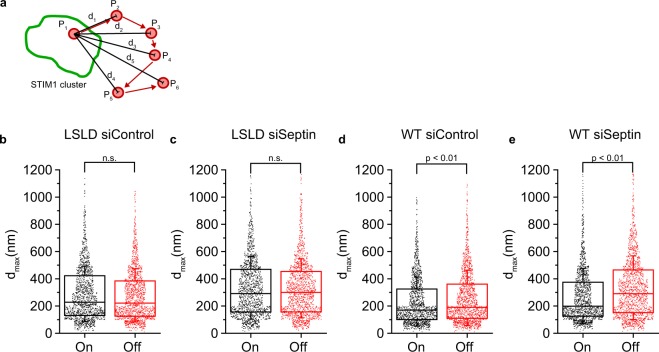
Maximum excursion of ORAI1 trajectories. (**a**) Representation of an ORAI1 trajectory (red dots and arrows, P_1_–P_6_) with its first localization within a STIM1-marked junction (outlined in green). The displacements *d*_1_–*d*_5_ from the first localization were calculated, and the largest displacement— *d*_5_ in this case— defined the maximum excursion for that trajectory. Maximum excursions were analyzed separately for tracks starting on or off STIM1-marked junctions. (**b**,**c**) Maximum observed excursion for ORAI1-LSLD trajectories starting within STIM1-marked junctions (On) or outside STIM1-marked junctions (Off), in siControl-treated and siSeptin4/5-treated cells, after TG stimulation. (**d**,**e**) Corresponding data for wildtype ORAI1. (**b**–**e**): *n* = 1500 trajectories for each condition. Statistical significance was determined using a two-tailed *t*-test.

Is there any kinetic barrier to ORAI1 movement out of STIM1-marked junctions? We compared ORAI1-LSLD trajectories starting within junctions, in siControl or siSeptin4/5 cells after TG, with those starting outside junctions in the same cells. Trajectories consisted of 6–25 50-ms steps and had a comparable distribution of durations under the different conditions examined [Supplementary Fig. 7]. We defined the excursion distance *d*_max_ as the largest displacement observed between the starting point and any point in the trajectory [Fig. 6a]. Depletion of septins 4/5 resulted in an increase in the median excursion of ORAI1-LSLD trajectories, with an equal effect on trajectories beginning within junctions and those beginning outside junctions [Fig. 6b,c]. Reinforcing this point, ORAI1-LSLD diffusion measured over the entire cell footprint in the MSD plot slowed after TG/EGTA treatment, and this global slowing was dependent on septin 4/5 [Supplementary Fig. 8; Table 3; Supplementary Movies 17, 18]. We conclude that septin-dependent barriers are in place across the cell footprint after TG treatment of control cells. The local barriers are not restricted to junctions, but they could nevertheless reduce the escape of free ORAI1 from junctions and favor rebinding of ORAI1 to STIM proteins under conditions where there is net exchange of ORAI1 out of the junctions.

**Table 3:** Diffusion estimates for ORAI1LSLD from single molecule tracking datasets.

ORAI1 LSLD	D [est1] µm^2^/sec	D [est2] µm^2^/sec
siControl Rest	0.089 ± 0.001	0.068 ± 0.001
siControl TG	0.065 ± 0.001	0.056 ± 0.001
siSeptin 4/5 Rest	0.112 ± 0.001	0.078 ± 0.001
siSeptin 4/5 TG	0.115 ± 0.001	0.074 ± 0.001

In parallel, we examined the excursion distances of wildtype ORAI1 trajectories at junctions and outside junctions after TG [Fig. 6d,e]. Although the experiment was intended only to check that we could see a difference in the distances with wildtype ORAI1, and was not explicitly designed for comparisons between wildtype ORAI1 and ORAI1-LSLD, it does offer a further perspective on ORAI1 confinement at junctions. The comparison of WT siSeptin to LSLD siSeptin, on junction, is a measure of purely STIM-dependent confinement. And the progressive decrease in excursion values *d*_max_ for LSLD siSeptin > LSLD siControl > WT siControl, on junction, indicates that both septins and STIM proteins contribute tangibly to confinement.

The constraints on ORAI1 excursion at junctions in Fig. 6d— comparing control cells on- and off-junction— may appear unexpectedly modest. This can be attributed primarily to local septin-dependent constraints on ORAI1 movement outside junctions, which are also evident with ORAI1-LSLD in Fig. 6b. However, another contributing factor could be that a fraction of junctional trajectories were incorrectly identified as nonjunctional [see above in the section ‘Single-molecule localization of ORAI1 channels in living cells’].

### STIM1 junctional area is reduced in siSeptin 4/5-treated cells

**Figure 7:**
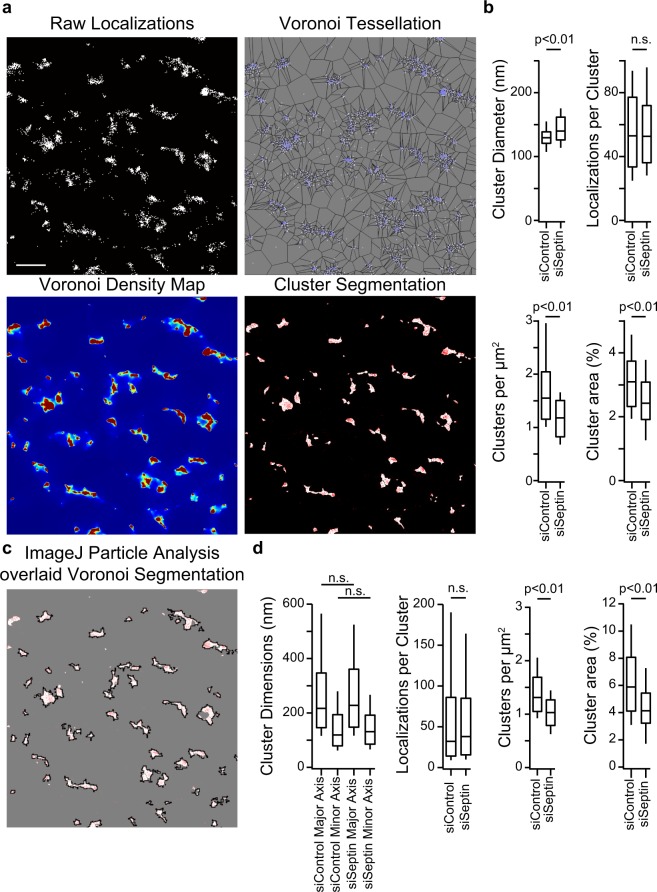
Quantitative analysis of STIM1 clustering after ER store depletion. (**a**) Example of Voronoi tessellation analysis. STIM1 localizations in a cell stimulated with TG are shown at top left, and, for the same region of interest, Voronoi cells from ClusterViSu analysis^42^, the corresponding Voronoi density map, and segmentation of the image into STIM1 clusters (white areas) together with the original localizations (red pixels). Scale bar is 1 μm. (**b**) STIM1-PATagRFP cluster diameters, molecules localized per cluster, clusters per μm^2^, and relative cell area occupied by clusters, after TG treatment, in siControl- and siSeptin4/5-treated cells. (**c**) Clusters defined by ImageJ particle analysis of the region in (**a**) are in close agreement with those defined by Voronoi tessellation. (**d**) STIM1 cluster dimensions, molecules localized per cluster, STIM1 clusters per μm^2^ and relative cell area occupied by STIM1 clusters from the ImageJ particle analysis. (**b**,**d**): siControl, n = 34; siSeptin, n = 35. Statistical significance was determined using a two-tailed *t*-test.

Finally, to assess whether septins 4/5 affect maintenance of the junctions themselves, we quantitated ER-PM junction numbers and areas in living cells under control and siSeptin4/5 conditions using STIM1 in store-depleted cells as a junctional marker. Recently published methods based on Voronoi tessellation have the advantage that they can analyze clustering directly from PALM localization data, thus avoiding the loss of resolution that comes with an intermediate step of converting localization data to an inferred image^41,42^. We conducted tessellation analysis of STIM1 localization data in living cells after TG using ClusterViSu^42^ [Fig. 7a,b]. The number of STIM1 molecules localized per cluster was unchanged in siSeptin4/5-treated cells compared to control cells, and cluster dimensions— expressed as equivalent diameter— were only modestly altered. However, the density of STIM1-marked clusters per μm^2^ in the image was significantly reduced by siSeptin4/5 treatment, and the median fraction of the cell footprint occupied by clusters was reduced by 23%, from 0.031 to 0.024.

The decrease in fractional junctional area was further confirmed by a separate ImageJ particle analysis, which estimated a reduction of 31%, from 0.059 to 0.041 [Fig. 7c,d]. ImageJ identified the same clusters as ClusterViSu in a given data set, but, perhaps related to the loss of resolution inherent in the processing steps, ImageJ tended to include a somewhat larger area within an individual assigned cluster, and also showed less tendency to subdivide contiguous areas into multiple clusters. It is unclear whether the finer subdivision performed by ClusterViSu corresponds to real substructure in the biological data sets. When we simulated data by placing localizations randomly within idealized 200-nm diameter or 400 nm-diameter circular junctions, ClusterViSu as implemented performed well on the 200-nm junctions but found apparent substructure in the 400-nm junctions where none was built into the simulated data [Supplementary Fig. 9]. The issue is tangential to our use of ClusterViSu, though, since both methods of clustering analysis supported the conclusion that there is a decrease in the number of clusters and the area occupied by STIM1 per μm^2^ in the cell footprint. Therefore, in addition to enhancing the retention of ORAI1 after it has arrived at an ER-PM junction, septins 4/5 also support the maintenance of a normal complement of ER-PM junctions where STIM1 and ORAI1 can interact.

## Discussion

**Figure 8:**
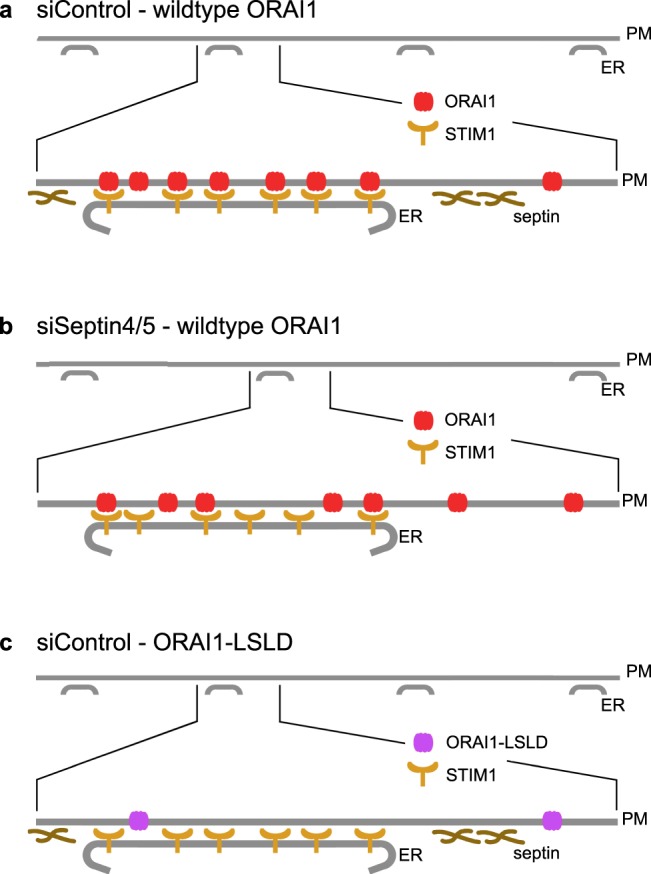
Cellular organization underlying STIM-ORAI Ca^2+^ signalling. (**a**) The basal condition in control cells expressing STIM1 and wildtype ORAI1. *Upper*, The ER maintains STIM1-competent junctions on the PM. *Lower*, Expanded view of a single junction after store depletion. ORAI1 has been recruited effectively and is engaged with STIM1. (**b**) siSeptin4/5-treated cells expressing STIM1 and wildtype ORAI1. The density of STIM1-competent ER-PM contacts is reduced. STIM1 still comes to the junctions, but ORAI1 is recruited less efficiently, and a lower fraction of junctional ORAI1 is engaged with STIM1. (**c**) Control cells expressing STIM1 and ORAI1-LSLD. STIM1 relocalizes to the junctions after store depletion, but ORAI1-LSLD is not recruited. This finding is the clearest evidence that septins and their associated proteins do not direct or confine ORAI1 to junctions, and implies the stronger conclusion that ORAI1 channels are recruited to ER-PM junctions solely by their interactions with STIM proteins.

Our findings highlight facets of PM organization that underlie cellular STIM-ORAI Ca^2+^ signalling, and identify control points that could coordinate regional changes in Ca^2+^ signalling in cells [Fig. 8]. The most prominent finding is that normal HeLa cells have a larger number of STIM1-competent junctional sites than septin4/5-depleted cells. Septins are known to organize and stabilize subcellular structures, and typically are visibly associated with those sites, for example with the mitotic spindle and the metaphase plate^43,44^ and with actin stress fibers^45^. In contrast, septin 4— taken as a marker for the class of septin filaments relevant to STIM-ORAI signalling— is not stably present at ER-PM junctions. The latter observation suggests that septins act through intermediaries to render the PM receptive to junction formation, to position ER tubules for junction formation, or to slow junction disassembly. The most plausible intermediaries would be cytoskeletal proteins^28^, given that cortical actin supports junction formation or stability^46^ and microtubules guide the continual reshaping of the ER^47–49^. The RNAi experiments here allowed us to infer global septin4/5-dependent processes that contribute to basal maintenance of junctions in cultured cells, but *in vivo* the same processes would be suited to coordinate more localized changes in STIM-ORAI signalling during the dynamic remodelling of ER-PM junctions that accompanies Ca^2+^ signalling, cell movement, and developmental changes in cellular architecture^50–52^.

A second intriguing finding is that STIM1 engages ORAI1 more effectively at ER-PM junctions in normal HeLa cells than in septin4/5-depleted cells. Individual junctions in control and septin4/5-depleted cells are comparable in size and recruit equivalent amounts of STIM1 after store depletion, and we showed previously that STIM1-ORAI1 gating— reflected in NFAT nuclear import triggered by the soluble STIM1 cytoplasmic domain— is unimpaired by septin4/5 depletion^10^. Together these observations imply that cells can modulate STIM-ORAI signalling efficiency by mechanisms that exert their effects inside the junctions. There is compelling indirect evidence of substructure within ER-PM junctions, with distinct junctional nanodomains dedicated to the assembly and the disassembly of STIM-ORAI complexes^53^. How and when septins speak to the internal structure of junctions is an important area for future investigation. One possibility is that a message is delivered when the junctions form, and that the junctions in normal HeLa cells are already poised for constructive STIM-ORAI engagement in their basal state. An alternative is that septin filaments or their associated proteins prime the junction for STIM-ORAI engagement when septins rearrange near junctions early in the response to ER Ca^2+^ store depletion^10^. The involvement of septins 4/5 once again raises the possibility that alterations in junctional signalling efficiency could be coordinated regionally and even very locally during physiological processes *in vivo*.

Finally, the generalized slowing of ORAI1 diffusion after global store depletion raises the possibility that Ca^2+^ signalling might be modulated locally or regionally by controlling ORAI1 diffusion itself. During physiological signalling that is restricted to only one part of the cell surface, ORAI1 diffusion might well be slowed within a zone of influence of a single store-depleted ER-PM junction or within a limited region in which ER stores have been depleted. Under these conditions ORAI1 diffusion into the zone or region would be greater than its slower diffusion out of the region. Assuming a localized change in *D* similar to what we have observed globally with TG, the ORAI1 concentration in the region would rise with time to a level 25–30% over the concentration outside the region, favoring increased STIM-ORAI interaction at junctions in the region if ORAI1 is otherwise limiting. From the diffusion equation, ORAI1 might be expected to accumulate from surrounding regions over a distance of one to a few micrometers on a time scale of a few seconds up to tens of seconds, a spatial and temporal range suited to modulating many localized cellular processes. For example, the lamellipodia of migrating endothelial cells exhibit small fluctuating Ca^2+^ signals on these distance and time scales and exhibit polarization of other Ca^2+^ signalling proteins toward the leading edge^54,55^. This polarization includes locally increased levels of STIM1 in the ER^55^, offering a precedent for a mechanism that modulates signalling by increasing local ORAI1 concentration.

Our single-molecule localization and tracking data establish that septins 4/5 at native levels facilitate STIM-ORAI Ca^2+^ signalling in several specific ways that do not require the continuing presence of septin structures at ER-PM junctions. Thus STIM-ORAI signalling is responsive to local or regional features imprinted by the septins. The shared dependence of cellular architecture, PM organization, and STIM-ORAI signalling on septins is likely to facilitate regional coordination of Ca^2+^ signalling with other signalling pathways and cellular processes.

## Methods

### DNA constructs and siRNA sequences

Full-length human STIM1, human ORAI1, human septin 4, GFP, PAGFP, and PATagRFP coding sequences were PCR-amplified and subcloned into the mammalian expression vectors pCMV (Addgene) or pMAX (Addgene). For pCMV-STIM1, the PAGFP or PATagRFP coding sequence was inserted in-frame following the STIM1 sequence. For pMAX-ORAI1 and pMAX-septin 4 the fluorescent protein sequence was inserted 5′ to the ORAI1 or septin 4 sequence. The ORAI1(L273S/L276D) plasmid containing, additionally, silent mutations (GGCCTGATCTTTATCGTCT to GGACTAATTTTCATTGTCT) in the siORAI1 target sequence was generated by PCR using Phusion® High-Fidelity DNA Polymerase (NEB). The full-length human septin 4 cDNA containing silent mutations in the siSEPT4#4 targeting sequence (GAACATCCAAGACAACCGA to GAACATCCAAGACAACAGG) was synthesized (IDT) and subcloned into mammalian expression vector pMAX (Addgene).

siRNA oligonucleotides were obtained from the Dharmacon Human siGenome collection (v2007 or 2010), with the exception of siControl, which was custom synthesized by Dharmacon. Sequences are listed in Supplementary Table 1.

### Cell culture and transfection

HeLa cells obtained from the American Type Culture Collection were cultured at 37 °C under 5% CO_2_ in Dulbecco’s Modified Eagle’s Medium (DMEM) containing 10% heat-inactivated fetal bovine serum (Omega Scientific). For single-particle tracking experiments, Hela cells in a 6-well plate were transfected with Mirus TransIT-LT1 (Mirus Bio) for 5–6 hours, adding 1.6–2.8 μg of total DNA per well in one of the following plasmid combinations:

0.8 μg STIM1-PAGFP + 1.0 μg PATagRFP-ORAI1 (or -ORAI1-LSLD)

0.8 μg unlabeled STIM1 + 1.0 μg PATagRFP-ORAI1 (or -ORAI1-LSLD) + 1.0 μg PAGFP-septin4

1.0 μg PAGFP-septin4 and 0.8 μg STIM1-PATagRFP

1.0 μg PAGFP-septin4 and 0.7 μg MAPPER

0.8 μg STIM1-PAGFP and 0.8 μg STIM1-PATagRFP.

Cells were seeded on poly-d-lysine treated Nunc™ Lab-Tek™ II Chambered Coverglass (Thermofisher Scientific) the following day. Briefly, Chambered Coverglass was coated with 100 μl per well of 0.1 mg/ml poly-d-lysine (Sigma-Aldrich) at 37 °C for 10 min followed by two washes with cold PBS. Experiments were performed 24 hours after cell seeding. Thapsigargin (TG) was purchased from Calbiochem. All other chemicals were purchased from Sigma.

For siRNA transfections, HeLa cells were reverse transfected with 25–50 nM siRNA using Mirus TransIT-X2 (Mirus Bio), with co-transfected fluorescence expression plasmid, according to the manufacturer’s instructions. siRNA-transfected cells were placed on coverglass after 48 hours. Cells were treated with siSeptin4/5 as indicated in the text. Cells were treated with the siRNA targeting native ORAI1 in all experiments where ORAI1-LSLD was expressed, in order to avoid assembly of heteromultimeric ORAI1-LSLD/ORAI1 channels containing wildtype ORAI1 subunits.

### Image acquisition

Cells were imaged in Ringer’s solution containing (in mM): NaCl 125, KCl 5, CaCl_2_ 1.5, MgCl_2_ 1.5, d-glucose 10, and HEPES/Na-HEPES 20 (pH 7.4). For depletion of Ca^2+^ stores, cells were imaged five minutes after switching the medium to 0-Ca^2+^ Ringer’s solution prepared with 1 μM TG and 1 mM EGTA (in mM): NaCl 125, KCl 5, MgCl_2_ 1.5, d-glucose 10, and HEPES/Na-HEPES 20 (pH 7.4).

Single particle tracking and photoactivated localization microscopy (sptPALM and PALM) were performed with a previously described microscope configuration^56^. Briefly, a Nikon Ti-E inverted microscope platform with a 100 × objective (CFI Apo TIRF oil immersion NA 1.49, Nikon) imaged HeLa cells held at 37 °C in a Tokai Hit Stage Top Incubator. Images were recorded with an Andor iXon 860E EMCCD camera and kept in focus with the Nikon Perfect Focus System. The excitation path 488 nm and 568 nm lasers (Genesis MX, Coherent) were air coupled with the 405 nm activation laser (CUBE, Coherent) and manually tuned for Total Internal Reflection Fluorescence (TIRF) excitation. PALM and sptPALM experiments were recorded with MetaMorph imaging software (Molecular Devices) at 20 Hz in streaming acquisition mode, in sequential acquisition periods of 25 s (500 frames) for the green/PAGFP channel, 25 s for the red/PATagRFP channel, and again 25 s for the green/PAGFP channel.

### Image analysis

#### Localizations

PALM localizations were generated with the ImageJ plugin ThunderSTORM. Images were filtered with a B-spline wavelet filter (order 3, scale 1.3–1.5), approximate molecular localizations assigned by a local maximum estimate with 8-connected neighborhoods, and sub-pixel localizations determined by weighted least-squares fitting to an integrated Gaussian PSF model over a 5 pixel radius with initial sigma value 1.3 pixels. Individual red-channel and green-channel PALM data were drift corrected to 100-nm TetraSpeck™ Microspheres (ThermoFisher Scientific), and the red and green channels were brought into register using the same fiducial markers. PALM localizations were visualized by the ‘Averaged Shifted Histogram’ command with lateral shift value 2 and magnification chosen to give a pixel size of 10 nm. The output representation was a 32-bit image with pixel size 10 nm.

#### Normalized colocalization

The colocalization and distance analyses were performed in MATLAB (MathWorks, Natick, MA). For colocalization analysis, output images from the PALM analysis were converted to binary images, and Normalized Colocalization in Green (NCG) was calculated as

$${\rm{NCG}}=({\rm{colocalized}}\,{\rm{pixels}}/{\rm{green}}\,{\rm{pixels}})/({\rm{red}}\,{\rm{pixels}}/{\rm{total}}\,{\rm{pixels}}).$$

#### Territories and median distances

PAGFP-septin 4 (or STIM1-PAGFP) PALM data from the two 25-s acquisitions in the green channel were merged, and filtered to remove localizations that did not fall within 100 nm of at least five other septin 4 (or STIM1) localizations. The binary green-channel image of the merged data defined the septin 4 (or STIM1) territory within the ROI, and was used to calculate the initial fractional coverage of red pixels in the corresponding binary red-channel image as

$${\rm{Fractional}}\,{\rm{coverage}}\,{\rm{of}}\,{\rm{red}}\,{\rm{pixels}}=({\rm{colocalized}}\,{\rm{pixels}}/{\rm{total}}\,{\rm{red}}\,{\rm{pixels}}).$$

The green territory was then dilated stepwise by three pixels at a time [Supplementary Fig. 10], recalculating the fractional coverage of red pixels at each step, until the fraction was equal to or greater than 50%. The total dilation at this point, in pixels, was converted to a median distance in nanometers for each region of interest (ROI) analyzed.

#### Trajectories

Single-molecule trajectories were analyzed with DiaTrack 3.05 software^57^. Image stacks were background-subtracted with the built-in ‘Subtract Background’ command and the data smoothed with a Gaussian filter (1.05 pixels). Subpixel localizations were determined by fitting to a Gaussian PSF model of half-width-half-max value 1.2 pixels, as described previously^56^. Particle selection ensured that molecules were sufficiently contrasted from their surroundings by using a value of 1.05 to remove blurred objects, and by applying user-determined intensity thresholds to remove objects that were too dim or too bright to be considered single fluorophores. After frame-by-frame single-molecule subpixel localization, trajectories were determined allowing a maximum displacement of three pixels (480 nm) between 50-ms frames and requiring a minimum trajectory lifetime of 3 frames, and drift corrected to 100-nm TetraSpeck™ Microspheres (ThermoFisher Scientific). Resulting track files were exported and processed in Igor Pro 6.2 (WaveMetrics).

#### MSD plots

The mean squared displacement (MSD) curve for each experimental condition was determined by averaging the squared displacement over all occurrences of the corresponding time intervals in all trajectories under that condition. The inclusion of overlapping steps in the calculation has been faulted, since it results in a correlated data set^32^, but this method is nonetheless commonly used to derive the MSD plot. (Note that the issue of overlapping steps does not arise in the single-step *D* estimates described below.) Error bars at each point represented the standard error of the mean. Corresponding ORAI1 displacement distributions were plotted for the 50, 100, and 150 ms time points for each experimental condition.

#### Diffusion coefficients

Average diffusion coefficients *D* for each protein and experimental condition were estimated in two ways. Estimate *D*_1_ was calculated from the mean squared displacement for a 50-ms interval (*MSD*_1_), corrected as required for the localization uncertainty estimated from STIM1 and ORAI1 fixed-sample controls^30^ and for the apparent contraction introduced by continuous data acquisition^29,31,32^. The basic equation relating *D* to expected mean squared displacement <*r*^2^> and time *τ* is

$$ < {r}^{2} > =4\,D\tau .$$

The correction for localization uncertainty^30^ is based on

$$\langle {r}_{observed}^{2}\rangle =\langle {r}_{step}^{2}\rangle +2{s}_{localization}^{2}$$

We estimated the contribution of localization error from STIM1 and ORAI1 ‘trajectories’ in fixed cells. Cells were fixed in 4% paraformaldehyde in phosphate-buffered saline for ten minutes at room temperature, washed, and imaged under the same experimental conditions as for living cells. The observed MSD values for a 50-ms interval in fixed cells (*MSD*_1,fixed_) were 0.0086 ± 0.0002 μm^2^ for STIM1 (from 15,350 trajectories) and 0.0085 ± 0.0002 μm^2^ for ORAI1 (from 11,436 trajectories). Thus 2*σ*_localization_^2^ ≈ 0.0085 μm^2^, corresponding to a two-dimensional localization error *σ* = 65 nm, or *σ*_x_ = *σ*_y_ = 46 nm.

The second correction is necessary because the tracked molecules are moving during continuous data acquisition. Fluorescence originating from intermediate positions along a step that starts at point X and arrives at point Y tends to make the step look shorter than the actual distance from X to Y. The correction factor when fluorescence data are collected continuously during a single step is multiplication by 1.5 [refs^29,31,32^]. Hence, incorporating both corrections, and for a 50-ms step,

1 $${D}_{1}=1.5\ast (MS{D}_{1}-MS{D}_{1,{\rm{fixed}}})/(4\ast 0.05\,{\rm{s}}).$$

Estimate *D*_2_ was the slope of the MSD curve between the 50-ms and 100-ms points, calculated from *MSD*_1_ and the mean squared displacement for a 100-ms interval (*MSD*_2_),

$${D}_{2}=(MS{D}_{2}-MS{D}_{1})/(4\ast 0.05\,{\rm{s}}).$$

### Inverse fluorescence recovery after photobleaching (iFRAP)

Modified iFRAP experiments were performed on HeLa cells 24–48 h after transfection with 1–2 μg of a plasmid encoding GFP-ORAI1 and an equal amount of a plasmid encoding unlabelled STIM1. Cells were imaged on a Zeiss LSM 800 confocal system with a 63x oil plan-apo 1.4 NA objective and a 488-nm laser. A selected region of each cell was imaged before TG treatment to obtain iFRAP data for nonjunctional ORAI1, and a selected ORAI1 cluster in a different region of the same cell was analyzed after TG treatment to obtain iFRAP data for junctional ORAI1. An area bracketing the selected ROI was bleached with laser illumination at high power, bringing the fluorescence signal close to background in the illuminated area, and bleaching laser illumination was continued during the subsequent 90 s, except during image collection at 1 s intervals. This procedure eliminated any signal increase in the clusters from newly recruited GFP-ORAI1, and allowed us to measure fluorescence loss due to GFP-ORAI1 exit from the ROI. A photobleaching control was run in parallel in each case, but no correction for photobleaching was required. Fluorescence data were recorded with Zeiss software, and statistical analysis was performed in Excel. Mean values (normalized and background corrected) were plotted from 3 independent experiments for each condition.

### Analyzing trajectories relative to ER-PM junctions

#### Trajectories at and away from junctions

For these analyses, both PAGFP and PATagRFP were localized and the localizations drift-corrected using DiaTrack 3.05 software. Localizations and trajectories were exported to MATLAB (MathWorks, Natick, MA), with each data set represented as a 2048 × 2048 array of 10-nm pixels, and the PAGFP array was aligned to the PATagRFP array via the fiducial TetraSpeck™ Microspheres. STIM1-marked ER-PM junctions were defined as regions where STIM1-PAGFP localized and clustered after TG treatment. STIM1 localizations that did not fall within 100 nm of at least five other STIM1 molecules were discarded, similarly to the density filtering performed in ThunderSTORM, described above. Gaps in ER-PM junction clusters were filled in with a 5-pixel (50-nm) dilation of each localization retained after density filtering. PATagRFP-ORAI1 tracks were then classed as “on junction” if the first localization in the track occurred in a STIM1-marked junction, and “off junction” if all localizations in the track were outside STIM1-marked junctions.

#### Excursion distances

Only tracks consisting of 6–25 steps were used for the analysis of excursion distances. The maximum excursion *d*_max_ was defined as the maximum displacement from the first localization to the furthest localization within the same track [Fig. 6a]. A total of 1500 tracks from each treatment group were randomly chosen and the *d*_max_ distributions were compared between conditions.

#### Diffusion coefficients at junctions and STIM-ORAI engagement

For each on-junction ORAI1 trajectory, the squared displacement (*r*_1_^2^) of the first 50-ms step was calculated based on the coordinates of the first and second localizations,

$${{r}_{1}}^{2}={({x}_{1}-{x}_{0})}^{2}+{({y}_{1}-{y}_{0})}^{2},$$

and the sample average *r*^2^ was calculated. *D* was determined from the averaged single-step *r*^2^ values as in [Equation 1], except that *MSD*_1_ was replaced by the averaged single-step *r*^2^ value. The fraction of junctional ORAI1 engaged with STIM1 was estimated based on the simplifying assumption that ORAI1 has only two diffusional states, as free ORAI1 (*D* given by the value for ORAI1-LSLD in siControl- or siSeptin4/5-treated cells after TG, as appropriate) or as STIM1-bound ORAI1 (*D* given by the measured STIM1 *D* value). Then

$${\rm{Fraction}}\,{\rm{of}}\,{\rm{ORAI}}1\,{\rm{engaged}}=({D}_{{\rm{LSLD}}}-{D}_{{\rm{WT}}})/({D}_{{\rm{LSLD}}}-{D}_{{\rm{STIM}}1}).$$

The single-step *D* values for ORAI1-LSLD on-junction were 0.0840 μm^2^ s^−1^ (siControl/TG) and 0.1298 μm^2^ s^−1^ (siSeptin/TG). Since STIM1 diffusion was not affected by siSeptin4/5 treatment, the average of STIM1 *D* values for siControl/TG and siSeptin/TG conditions, 0.0394 μm^2^ s^−1^, was used.

### STIM1 clustering analysis

Clustering of PALM localizations were analyzed with ClusterVisu Voronoi tessellation segmentation software^42^. The localization file from ThunderSTORM was imported, and at least three smaller ROIs (~16–20 µm^2^) were chosen per cell to increase the analysis speed. ClusterViSu uses Monte Carlo simulations— here set at 100 iterations— to determine the expected Voronoi polygon area distribution for a random data set consisting of the same number of localizations in the same ROI, in order to define a threshold value of cell area below which individual Voronoi cells are accepted as part of a nonrandom cluster. A Voronoi density map with 10-nm pixel size was constructed on this basis. Watershed segmentation was applied to this map, and clusters less than 10 pixels in size were excluded. Statistics on cluster dimensions, localizations per cluster, and cluster density per unit area were collected and analyzed in Excel.

As an independent check, clusters were analyzed using ImageJ ‘Analyze Particles’ to determine cluster sizes, localizations per cluster, and cluster density per unit area. The same ROIs were used and localization images reconstructed similarly with a 10-nm pixel size.

### Cluster simulation

Randomized clusters were generated using a custom program written in MATLAB (MathWorks, Natick, MA). First, 17 cluster centers were placed randomly in a 4 μm x 4 μm region of interest. Then either 32 or 100 localizations, each with 70-nm localization uncertainty, were placed randomly in a 200-nm or 400-nm circular disc surrounding each cluster center, and background noise localizations amounting to 0.7% of pixels in the ROI was added randomly. The numbers of localizations per cluster, localization uncertainty, and level of background localizations were chosen to be representative of the actual data in our experiments. The generated clusters were then imported to ClusterVisu for analysis as described above.

### Statistics

Statistical analysis was carried out in MATLAB. Comparisons in Figs 1, 3, 4, 5 and 6 were by *t*-test, with equal or unequal variance as determined by an F-test. The box plot whiskers represent 1 SD from the mean. Comparisons in Fig. 7 were by Student’s *t*-test, carried out in Excel, and the box plots were drawn in Igor Pro 6.2. Box-plot whiskers represent the 10%–90% range of observed values. In the MSD plots and the intensity decay curves of the iFRAP experiments, error bars indicate SEM.

## Supplementary information

Supplementary Information

Supplementary Movie 1. ORAI1 movement relative to STIM1 in resting siControl cells

Supplementary Movie 2. ORAI1 movement relative to STIM1 after TG stimulation in siControl cells.

Supplementary Movie 3. ORAI1 movement relative to STIM1 in resting siSeptin 4/5 cells.

Supplementary Movie 4. ORAI1 movement relative to STIM1 after TG stimulation in siSeptin 4/5 cells.

Supplementary Movie 5. MAPPER localization relative to septin 4 in resting cells.

Supplementary Movie 6. MAPPER localization relative to septin 4 after TG stimulation.

Supplementary Movie 7. STIM1 movement relative to septin 4 in resting cells.

Supplementary Movie 8. STIM1 movement relative to septin 4 after TG stimulation.

Supplementary Movie 9. Septin 4 movement relative to STIM1 in resting cells.

Supplementary Movie 10. Septin 4 movement relative to STIM1 after TG stimulation.

Supplementary Movie 11. ORAI1-LSLD movement relative to STIM1 in resting cells.

Supplementary Movie 12. ORAI1-LSLD movement relative to STIM1 after TG stimulation.

Supplementary Movie 13. ORAI1 movement relative to septin 4 in resting cells.

Supplementary Movie 14. ORAI1 movement relative to septin 4 after TG stimulation.

Supplementary Movie 15. ORAI1-LSLD movement relative to septin 4 in resting cells.

Supplementary Movie 16. ORAI1-LSLD movement relative to septin 4 after TG stimulation.

Supplementary Movie 17. ORAI1-LSLD movement relative to STIM1 in resting siSeptin 4/5 cells.

Supplementary Movie 18. ORAI1-LSLD movement relative to STIM1 after TG stimulation in siSeptin 4/5 cells.

## Data Availability

Data are available from the corresponding author upon request.

## Code Availability

Custom MATLAB codes are available from Dr Chen Zhang or the corresponding author upon request.
