# Supplementary material for: Septins organize endoplasmic reticulum-plasma membrane junctions for STIM1-ORAI1 calcium signalling
Source: Sci Rep. 2019 Jul 25;9:10839. doi: 10.1038/s41598-019-46862-w (PMC6658532; doi:10.1038/s41598-019-46862-w)
Supplement: Supplementary file 1 — Supplementary Information [file 41598_2019_46862_MOESM1_ESM.pdf]

Septins organize endoplasmic reticulum-plasma membrane junctions for STIM1-ORAI1 calcium signalling

Zachary B Katz[1,2,6], Chen Zhang[1,6], Ariel Quintana[1,5], Björn F Lillemeier[2,*], Patrick G Hogan[1,3,4,*]

[1] Division of Signalling and Gene Expression, La Jolla Institute for Immunology, La Jolla, CA 92037, USA

[2] NOMIS Center for Immunobiology and Microbial Pathogenesis & Waitt Advanced Biophotonics Center, Salk Institute for Biological Studies, La Jolla, CA 92037, USA

[3] Program in Immunology, University of California San Diego, La Jolla, CA 92037, USA

[4] Moores Cancer Center, University of California San Diego, La Jolla, CA 92093, USA

[5] Present address: Translational Science Division, Clinical Science Department, Moffitt Cancer Center Magnolia Campus, Tampa, FL 33612, USA

[6] These authors contributed equally: Zachary B Katz, Chen Zhang
[*] email: blillemeier@salk.edu, phogan@lji.org

SUPPLEMENTARY FIGURE LEGENDS

Supplementary Figure 1. PATagRFP-ORAI1 in a resting cell. ORAI1 (magenta) was broadly distributed over the footprint of resting cells. This image encompasses a larger region of the HeLa cell in Figure 1a. STIM1-PAGFP (green) was visible in near-PM ER of resting cells.

Supplementary Figure 2. STIM1-STIM1 overlap. STIM1-STIM1 overlap was assessed in the same way as for ORAI1-STIM1 overlap in Figure 1b. (a) Representative 16.7 μm x 16.7 μm region of a cell after TG stimulation. STIM1-PAGFP data were acquired during a 25-s window, and STIM1-PATagRFP data during a following 25-s window [see Methods]. Pixel coloring indicates that STIM1 was localized to the pixel during the first period (green), the second period (magenta), or both periods (white). (b,c) Normalized colocalization and median distance analyses for the STIM1-STIM1 comparison, as in Figure 1c and 1d for the ORAI1-STIM1 comparison.

(b),(c): Data from 2 independent experiments; Rest, 27 regions from 9 cells; TG, 24 regions from 8 cells. Statistical significance was determined using a two-tailed *t*-test.

Supplementary Figure 3. Histograms of ORAI1 displacements at 50 ms, 100 ms, and 150 ms. (a) For siControl-treated cells, the histogram shifted to smaller displacements after TG stimulation (red bars) than at rest (black bars). (b) For siSeptin4/5-treated cells, the difference was almost imperceptible. (c) The shift was restored in siSeptin4/5-treated cells reconstituted with RNAi-resistant septin 4. The 50-ms data in (a)-(c) are the same as in Figure 1p. (d) Small apparent displacements of immobilized ORAI1 in fixed cells (grey bars) are due to localization uncertainty. The 50-ms data for TG-stimulated cells (red bars) are replotted from (a) for comparison with this limiting case.

Supplementary Figure 4. A visual estimate of the STIM1 radius of confinement. MSD data for STIM1 after TG (black curve) are replotted from Figure 2c, along with the theoretical linear MSD plot (red line) for unconstrained diffusion with diffusion coefficient D estimated from the observed mean r^2 value at 50 ms. The theoretical MSD plot is offset at time 0 to account for the estimated localization

uncertainty of 65 nm— corresponding r^2, 0.0085 μm^2— and for the 'blurring' caused by continuous data acquisition throughout the 50-ms frame [see Methods]. The plateau that would be reached by molecules constrained to an equivalent confinement radius r_c of 250 nm is indicated. Given the continuing upward slope of the MSD plot, the observed data are likely to represent the weighted sum of the average r^2 values of a diffusing population of unconfined STIM1 molecules or STIM1 molecules escaping confinement and the effectively constant average r^2 values of a confined population characterized by an early plateau. In this case the plateau and the radius of confinement for the confined subpopulation would be even lower.

Supplementary Figure 5. Septin 4 territories. Septin 4 localized in the 25 s prior to (green) and in the 25 s after (red) tracking ORAI1 defines septin 4 territories and, conversely, regions largely devoid of septin 4. Bleaching of PAGFP-septin 4 that was present in the TIRF layer during the first period of data collection may conceivably have led to overrepresentation of sites with newly added septin 4 in the localizations acquired during the second period of data collection.

Supplementary Figure 6. Septin 4 territories and ER-PM junctions. (a,b) Localizations of PAGFP-septin 4 (green) and PATagRFP-MAPPER (magenta) in a resting cell and in a TG-stimulated cell. (c,d) Localizations of PAGFP-septin 4 (green) and STIM1-PATagRFP (magenta) in a resting cell and in a TG-stimulated cell. The images illustrate the segregation of septin 4 territories from ER-PM junctions over larger regions than those depicted in Figure 3.

Supplementary Figure 7. ORAI1-LSLD and ORAI1 trajectory lengths. The histograms verify that the distributions of trajectory lengths were similar for the data sets compared in Figure 6. Black bars, trajectories starting on junction; red bars, trajectories starting off junction.

Supplementary Figure 8. MSD plots of ORAI1-LSLD molecules under the conditions indicated. siControl, Rest, 144,258 trajectories in 35 cells; siControl, TG, 170,600 trajectories in 46 cells; siSeptin4/5, Rest, 139,535 trajectories in 30 cells; siSeptin4/5, TG, 183,053 trajectories in 35 cells. Error bars indicate SEM.

Supplementary Figure 9. ClusterViSu analysis of simulated data. Thirty-two or 100 localizations, as indicated, each with 70-nm localization uncertainty, were placed randomly in seventeen 200-nm or 400-nm circular discs; and then the discs themselves were placed randomly in a 4 μm x 4 μm region of interest [see Methods]. ClusterViSu analysis was performed as for Figure 7a. ClusterViSu as implemented performed well in recognizing the individual 200-nm discs, but found substructure in the 400-nm discs.

Supplementary Figure 10. Measuring the median distance from localizations of ORAI1 to a STIM1 territory. The example is based on Figure 1b, reproduced at left. In the first step of the analysis (Original), compact territories characterized by a sufficient density of STIM1-PAGFP localizations (grey, enclosed by yellow boundaries) were defined as described in Methods. In subsequent steps, the territories were dilated stepwise by three pixels (30 nm). Only a few of the resulting dilated territories (Dilation x 1, Dilation x 5, Dilation x 8) are depicted. The median distance was that of the first dilation that covered at least 50% of the PATagRFP-ORAI1 localizations. The same approach was used to determine the median distance from localizations of ORAI1 to septin territories.

SUPPLEMENTARY MOVIE LEGENDS

Supplementary Movie 1. ORAI1 movement relative to STIM1 in resting siControl cells. 3 µm x 3 µm regions from different cells depicting PATagRFP-ORAI1 (magenta) single molecule movement registered to STIM1-PAGFP PALM data (green). Scale bars = 500 nm and the movies play at 20 frames per second (real-time).

Supplementary Movie 2. ORAI1 movement relative to STIM1 after TG stimulation in siControl cells. 3 µm x 3 µm regions from different cells depicting PATagRFP-ORAI1 (magenta) single molecule movement registered to STIM1-PAGFP PALM data (green). Scale bars = 500 nm and the movies play at 20 frames per second (real-time).

Supplementary Movie 3. ORAI1 movement relative to STIM1 in resting siSeptin 4/5 cells. 3 µm x 3 µm regions from different cells depicting PATagRFP-ORAI1 (magenta) single molecule movement registered to STIM1-PAGFP PALM data (green). Scale bars = 500 nm and the movies play at 20 frames per second (real-time).

Supplementary Movie 4. ORAI1 movement relative to STIM1 after TG stimulation in siSeptin 4/5 cells. 3 µm x 3 µm regions from different cells depicting PATagRFP-ORAI1 (magenta) single molecule movement registered to STIM1-PAGFP PALM data (green). Scale bars = 500 nm and the movies play at 20 frames per second (real-time).

Supplementary Movie 5. MAPPER localization relative to septin 4 in resting cells. 3 µm x 3 µm regions from different cells depicting PATagRFP-MAPPER (magenta) single molecule movement registered to PAGFP-septin 4 PALM data (green). Scale bars = 500 nm and the movies play at 20 frames per second (real-time).

Supplementary Movie 6. MAPPER localization relative to septin 4 after TG stimulation. 3 µm x 3 µm regions from different cells depicting PATagRFP-MAPPER (magenta) single molecule movement

registered to PAGFP-septin 4 PALM data (green). Scale bars = 500 nm and the movies play at 20 frames per second (real-time).

Supplementary Movie 7. STIM1 movement relative to septin 4 in resting cells. 3 µm x 3 µm regions from different cells depicting STIM1-PATagRFP (magenta) single molecule movement registered to PAGFP-septin 4 PALM data (green). Scale bars = 500 nm and the movies play at 20 frames per second (real-time).

Supplementary Movie 8. STIM1 movement relative to septin 4 after TG stimulation. 3 µm x 3 µm regions from different cells depicting STIM1-PATagRFP (magenta) single molecule movement registered to PAGFP-septin 4 PALM data (green). Scale bars = 500 nm and the movies play at 20 frames per second (real-time).

Supplementary Movie 9. Septin 4 movement relative to STIM1 in resting cells. 3 µm x 3 µm regions from different cells depicting PATagRFP-septin 4 (magenta) single molecule movement registered to STIM1-PAGFP PALM data (green). Scale bars = 500 nm and the movies play at 20 frames per second (real-time).

Supplementary Movie 10. Septin 4 movement relative to STIM1 after TG stimulation. 3 µm x 3 µm regions from different cells depicting PATagRFP-septin 4 (magenta) single molecule movement registered to STIM1-PAGFP PALM data (green). Scale bars = 500 nm and the movies play at 20 frames per second (real-time).

Supplementary Movie 11. ORAI1-LSLD movement relative to STIM1 in resting cells. 3 µm x 3 µm regions from different cells depicting PATagRFP-ORAI1LSLD (magenta) single molecule movement registered to STIM1-PAGFP PALM data (green). Scale bars = 500 nm and the movies play at 20 frames per second (real-time).

Supplementary Movie 12. ORAI1-LSLD movement relative to STIM1 after TG stimulation. 3 µm x 3 µm regions from different cells depicting PATagRFP-ORAI1LSLD (magenta) single molecule

movement registered to STIM1-PAGFP PALM data (green). Scale bars = 500 nm and the movies play at 20 frames per second (real-time).

Supplementary Movie 13. ORAI1 movement relative to septin 4 in resting cells. 3 µm x 3 µm regions from different cells depicting PATagRFP-ORAI1 (magenta) single molecule movement registered to PAGFP-septin 4 PALM data (green). Scale bars = 500 nm and the movies play at 20 frames per second (real-time).

Supplementary Movie 14. ORAI1 movement relative to septin 4 after TG stimulation. 3 µm x 3 µm regions from different cells depicting PATagRFP-ORAI1 (magenta) single molecule movement registered to PAGFP-septin 4 PALM data (green). Scale bars = 500 nm and the movies play at 20 frames per second (real-time).

Supplementary Movie 15. ORAI1-LSLD movement relative to septin 4 in resting cells. 3 µm x 3 µm regions from different cells depicting PATagRFP-ORAI1LSLD (magenta) single molecule movement registered to PAGFP-septin 4 PALM data (green). Scale bars = 500 nm and the movies play at 20 frames per second (real-time).

Supplementary Movie 16. ORAI1-LSLD movement relative to septin 4 after TG stimulation. 3 µm x 3 µm regions from different cells depicting PATagRFP-ORAI1LSLD (magenta) single molecule movement registered to PAGFP-septin 4 PALM data (green). Scale bars = 500 nm and the movies play at 20 frames per second (real-time).

Supplementary Movie 17. ORAI1-LSLD movement relative to STIM1 in resting siSeptin 4/5 cells. 3 µm x 3 µm regions from different cells depicting PATagRFP-ORAI1LSLD (magenta) single molecule movement registered to STIM1-PAGFP PALM data (green). Scale bars = 500 nm and the movies play at 20 frames per second (real-time).

Supplementary Movie 18. ORAI1-LSLD movement relative to STIM1 after TG stimulation in siSeptin 4/5 cells. 3 µm x 3 µm regions from different cells depicting PATagRFP-ORAI1LSLD

(magenta) single molecule movement registered to STIM1-PAGFP PALM data (green). Scale bars = 500 nm and the movies play at 20 frames per second (real-time).

Katz_etal_fig. S1

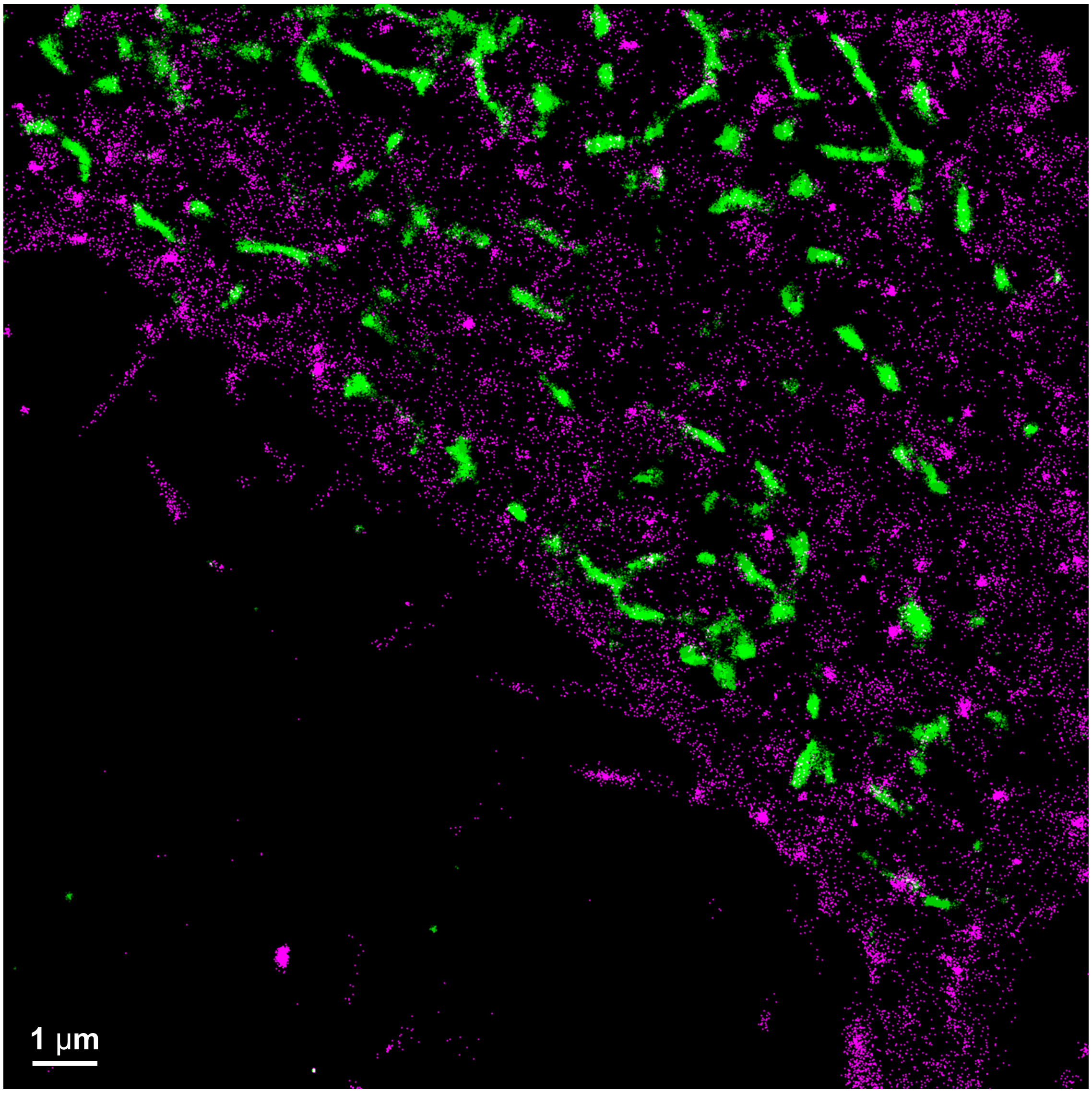

Katz_etal_fig. S2

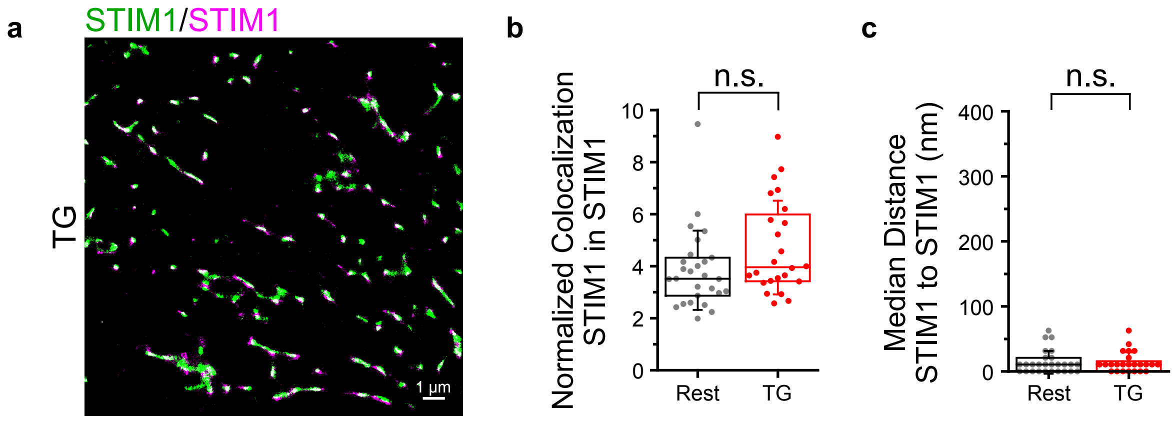

Katz_etal_fig. s3

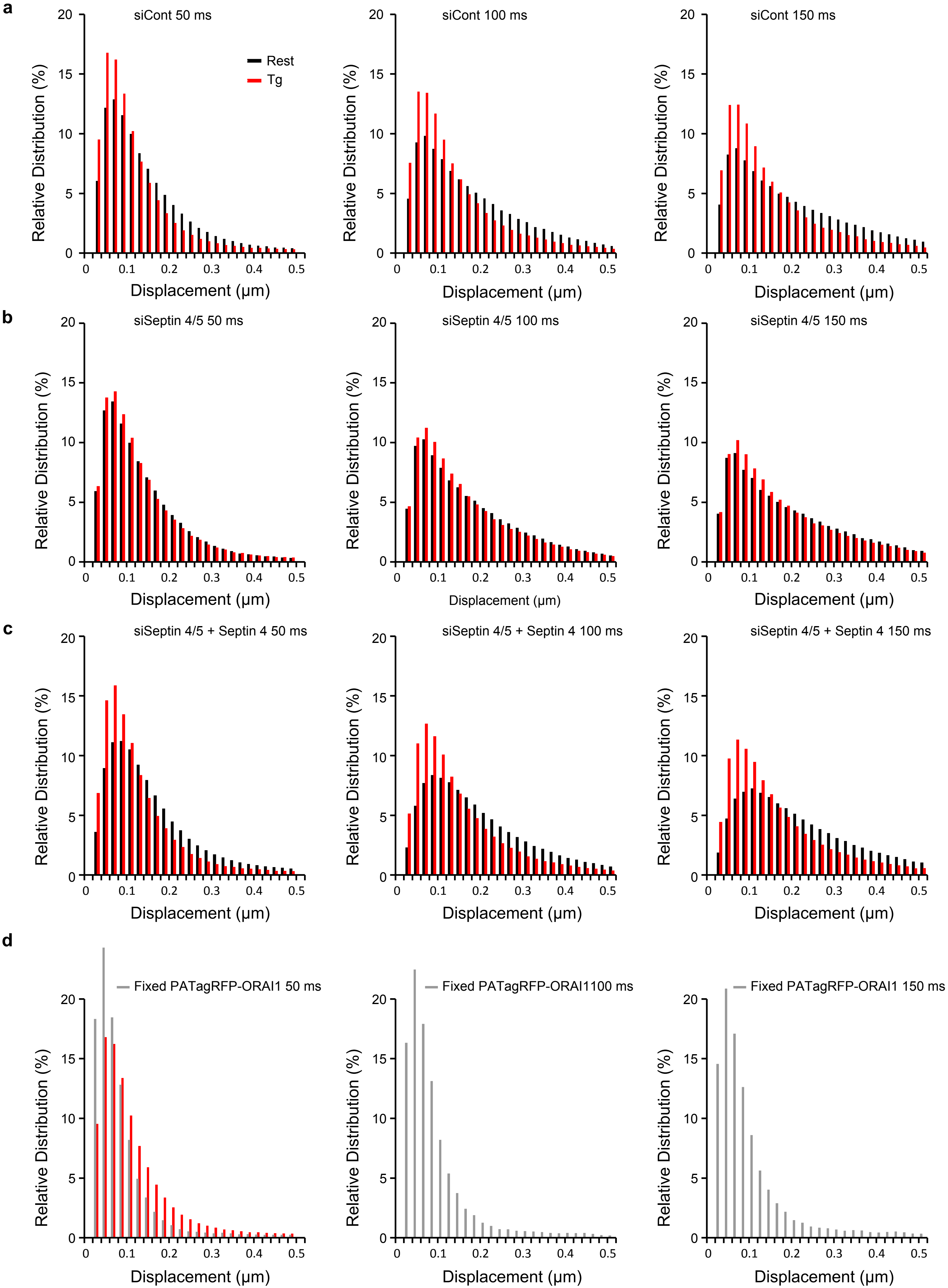

Katz_etal_fig. s4

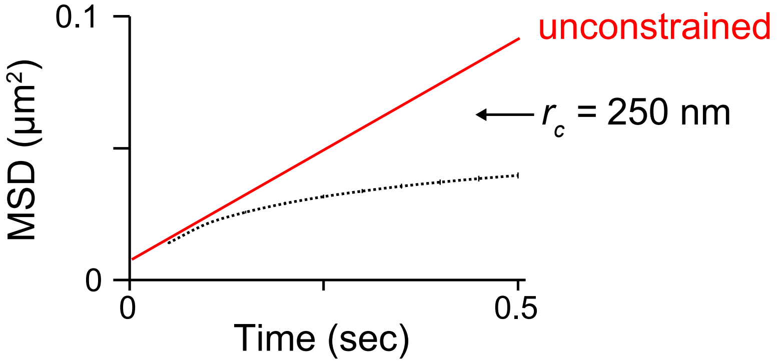

Katz_etal_fig. s5

Septin 4 before ORAI1 tracking / Septin 4 after ORAI1 tracking

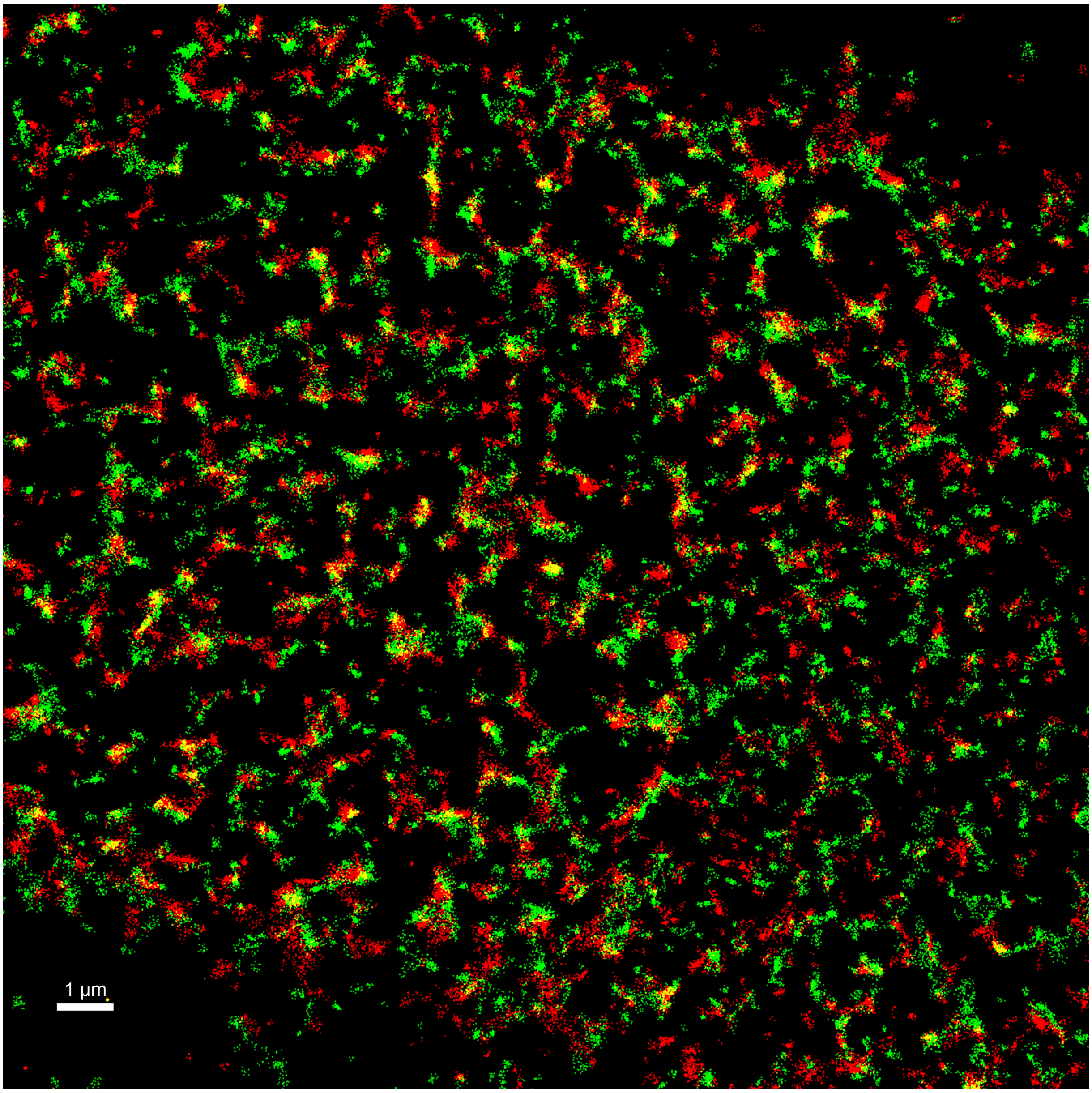

Katz_etal_fig. s6

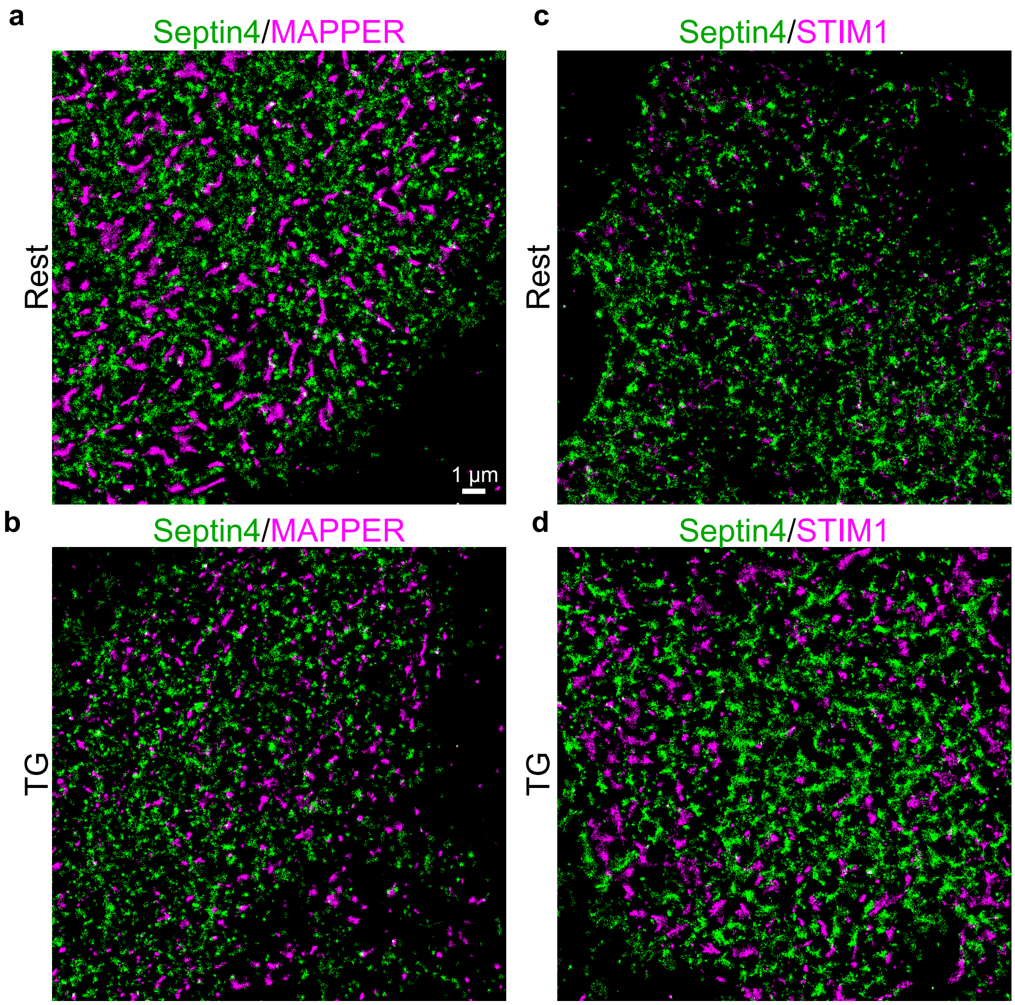

Katz_etal_fig. S7

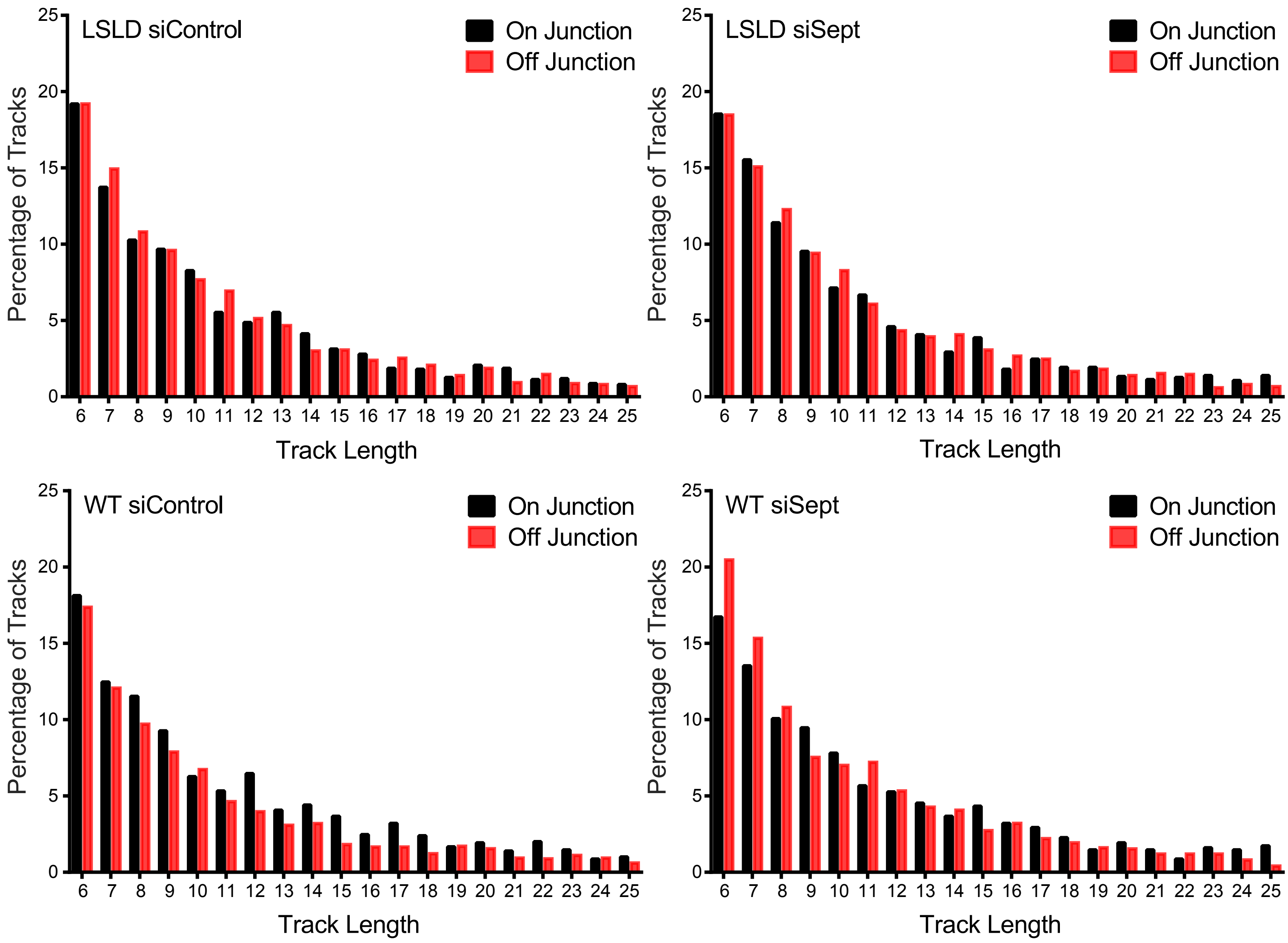

Katz_etal_fig. s8

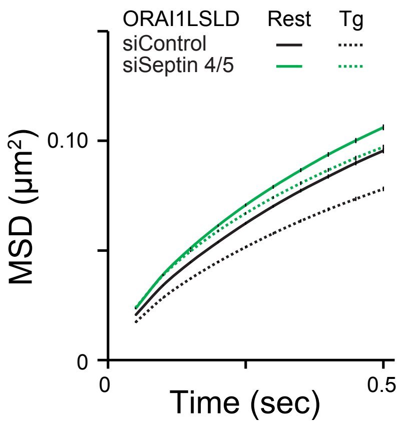

Katz_etal_fig. s9

100 localizations in 200 nm diameter

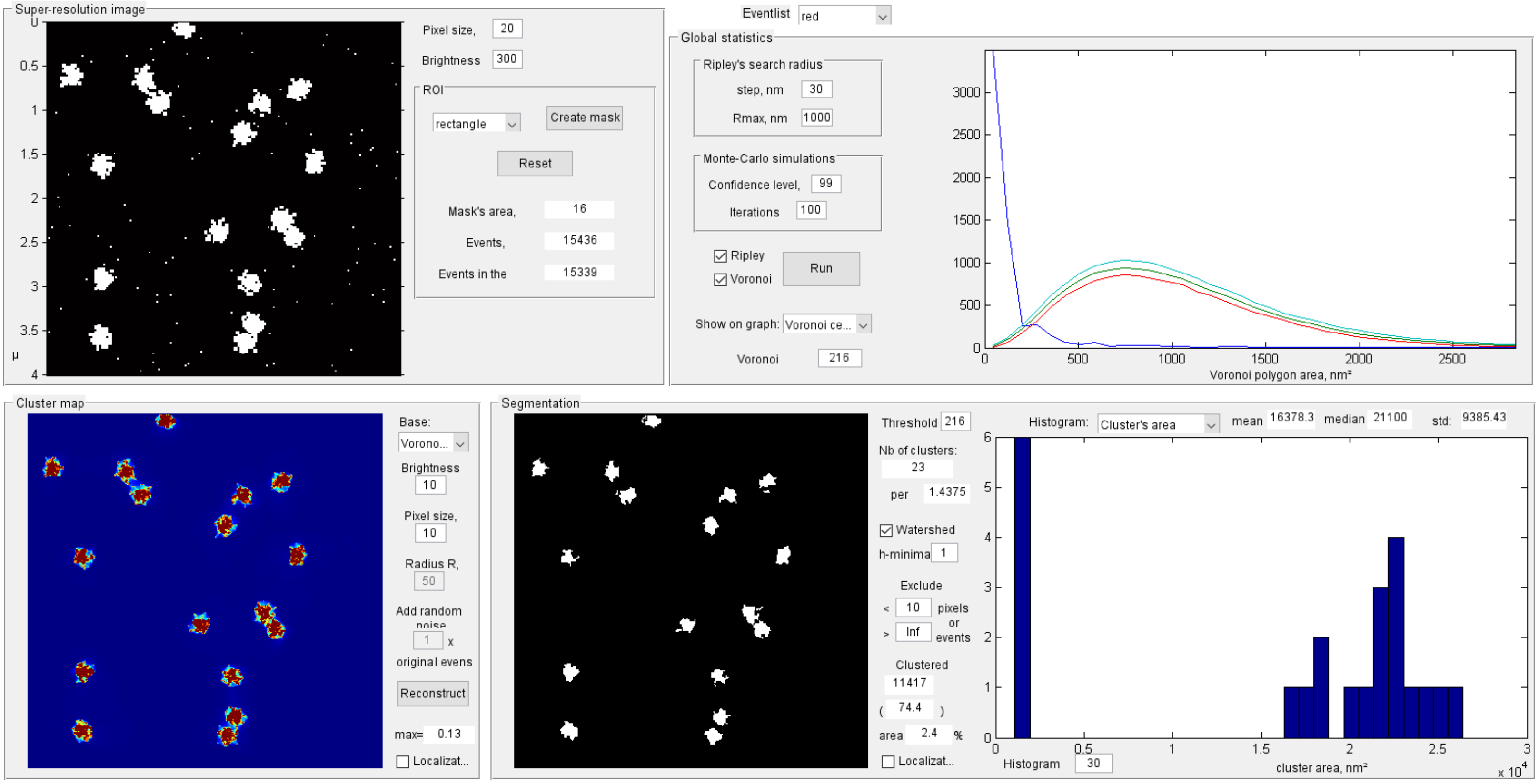

32 localizations in 200 nm diameter

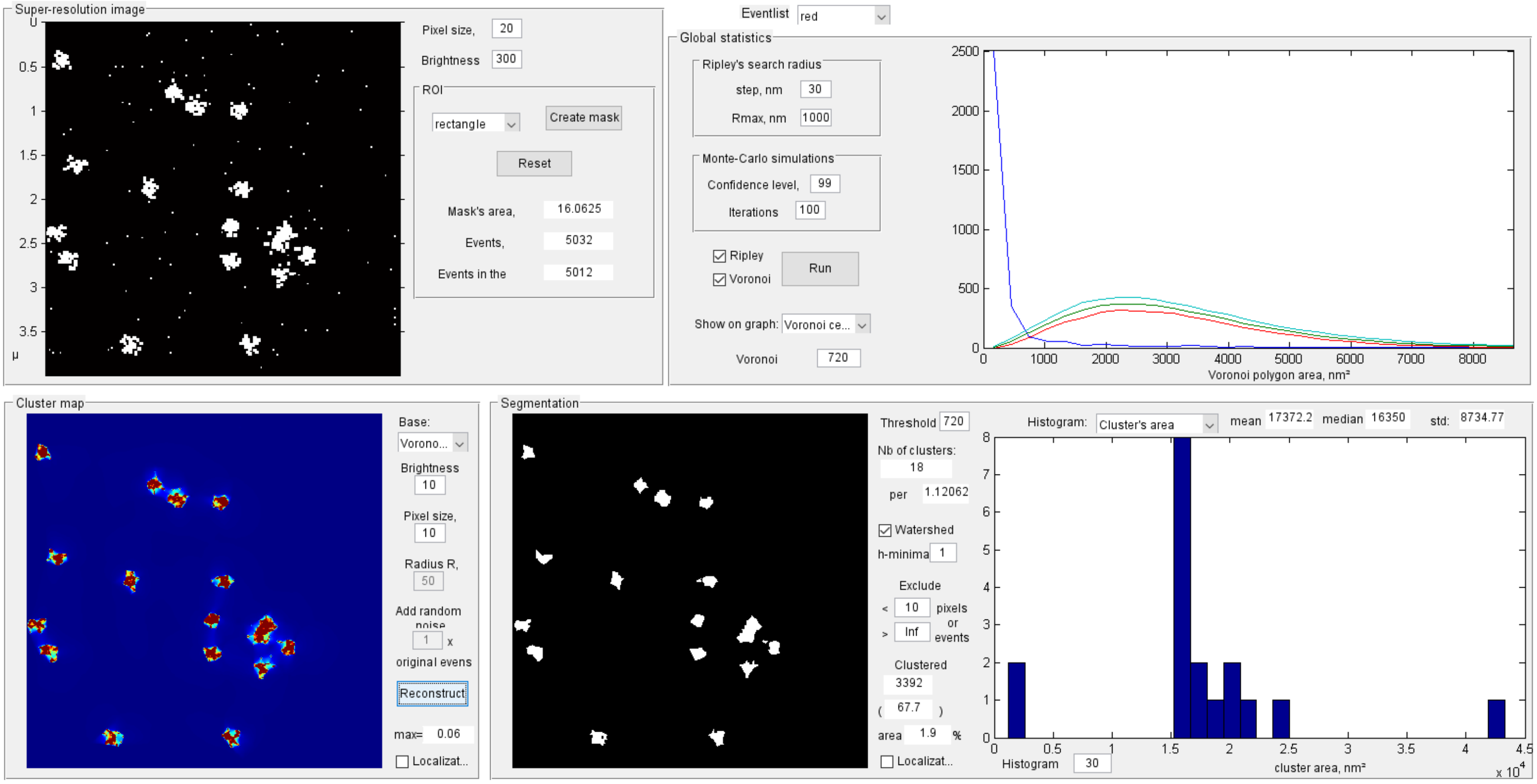

Katz_etal_fig. s9

100 localizations in 400 nm diameter

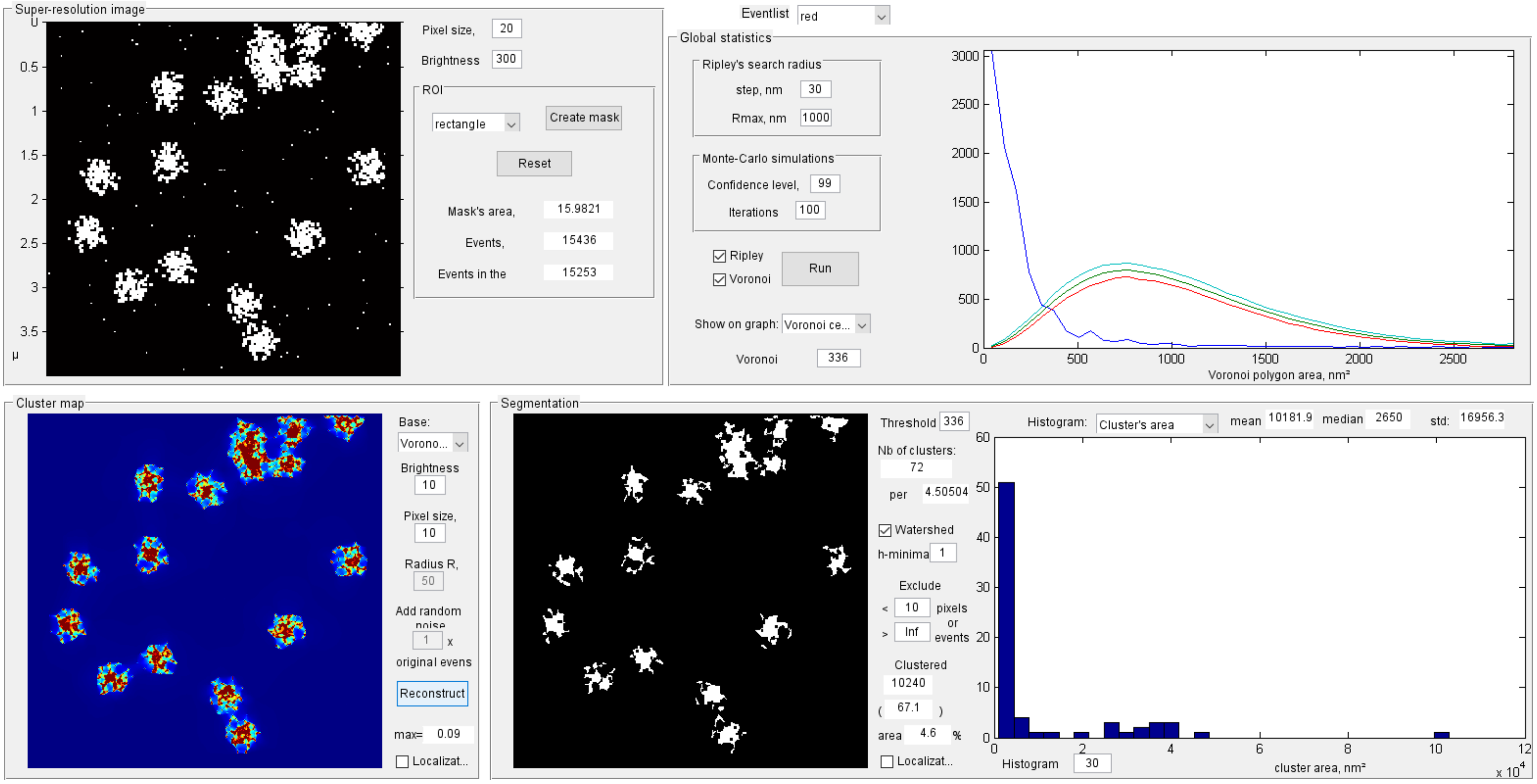

32 localizations in 400 nm diameter

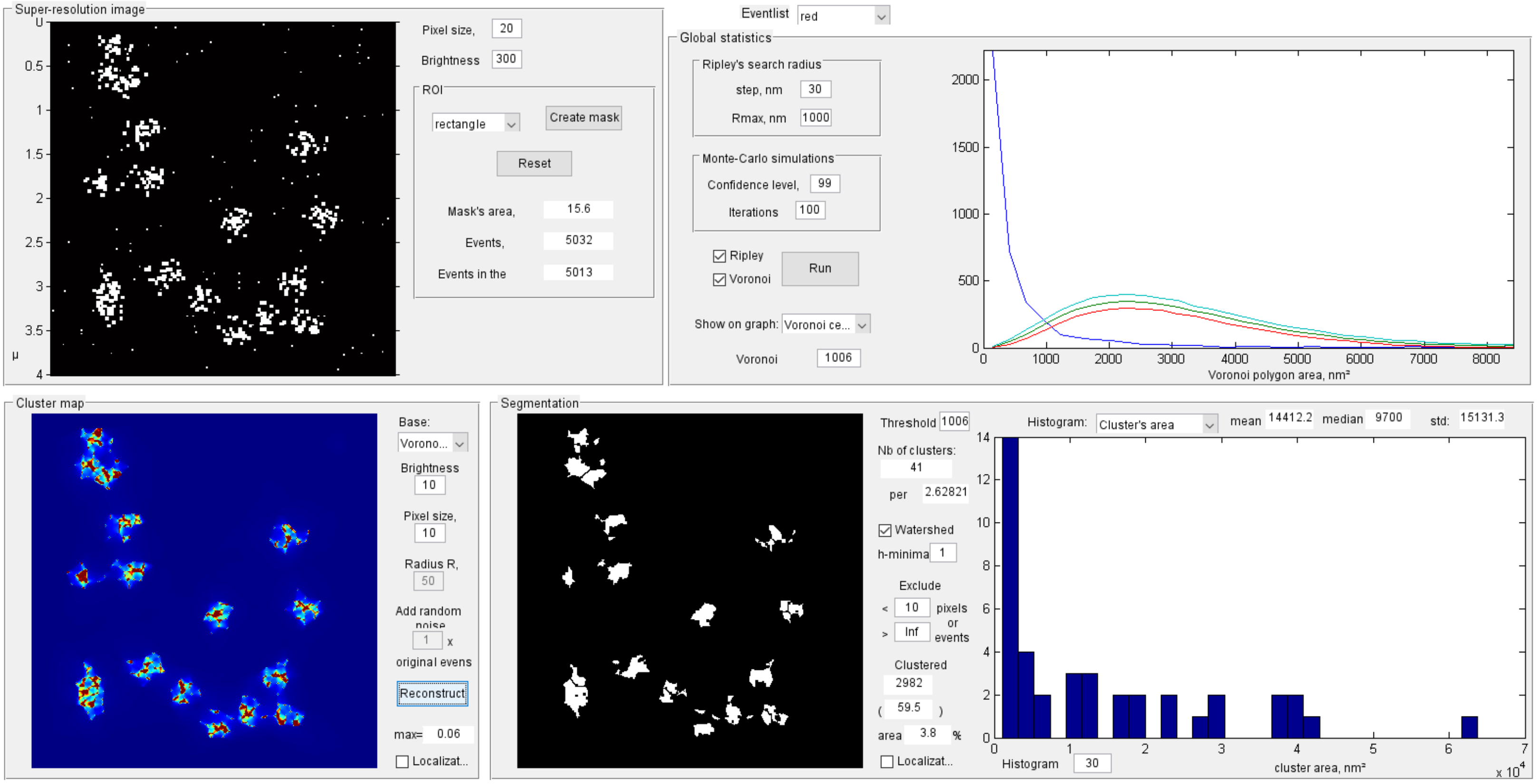

Katz_etal_fig. s10

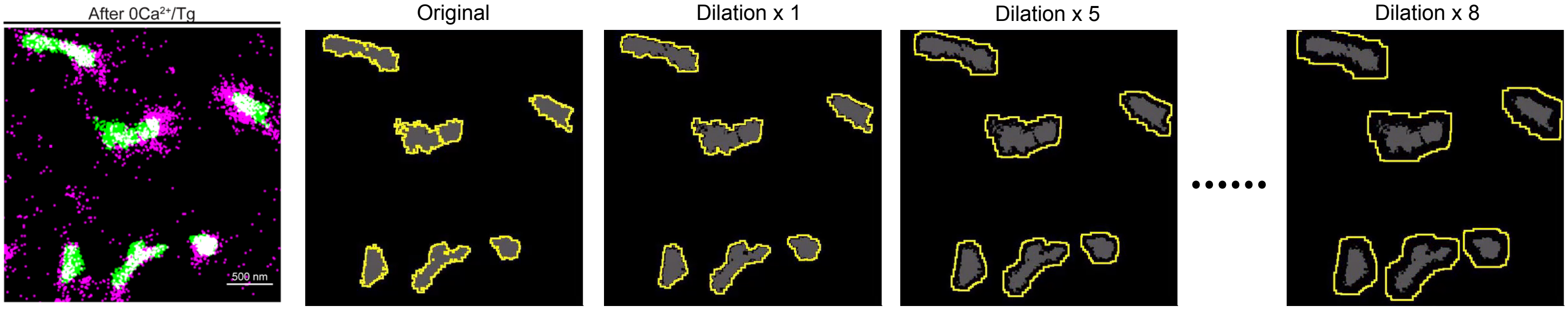

SUPPLEMENTARY TABLE

Supplementary Table 1: siRNA sequences

Gene symbol	EntrezGene ID	siRNA_sense
siControl	N/A	GCUAUUGCAUGUCGAAAUA
siORAI1	84876	GGCCUGAUCUUUAUCGUCU
siSTIM1	6786	GGUGGUGUCUAUCGUUAUU
siSEPT4#4	5414	GAACAUCCAAGACAACCGA
